# Faunal biodiversity of the lower abyssal and hadal zones of the Japan, Ryukyu and Izu-Ogasawara trenches (NW Pacific Ocean; 4534-9775 m)

**DOI:** 10.3897/BDJ.14.e182172

**Published:** 2026-03-03

**Authors:** Alan J. Jamieson, Denise J.B. Swanborn, Todd Bond, Megan C. Cundy, Yoshihiro Fujiwara, Dhugal Lindsay, Melanie S. Stott, Hiroshi Kitazato

**Affiliations:** 1 Minderoo-UWA Deep-Sea Research Centre, School of Biological Sciences and Oceans Institute, The University of Western Australia, Perth, Australia Minderoo-UWA Deep-Sea Research Centre, School of Biological Sciences and Oceans Institute, The University of Western Australia Perth Australia; 2 Research Institute for Global Change (RIGC), Japan Agency for Marine-Earth Science and Technology (JAMSTEC), Yokosuka, Japan Research Institute for Global Change (RIGC), Japan Agency for Marine-Earth Science and Technology (JAMSTEC) Yokosuka Japan; 3 X-STAR, Japan Agency for Marine-Earth Science and Technology (JAMSTEC), Yokosuka, Japan X-STAR, Japan Agency for Marine-Earth Science and Technology (JAMSTEC) Yokosuka Japan; 4 Tokyo University of Marine Science and Technology, School of Marine Resources and Environment, and regional hub of the Danish Center for Hadal Research at Tokyo, Tokyo, Japan Tokyo University of Marine Science and Technology, School of Marine Resources and Environment, and regional hub of the Danish Center for Hadal Research at Tokyo Tokyo Japan

**Keywords:** biodiversity, benthos, hadal zone, deep sea, imaging, ID guide

## Abstract

*In-situ* video-based observations collected using multiple platforms are increasingly used for biodiversity assessments at abyssal and hadal depths, sometimes complemented by physical sampling. Here, we present an imagery-based guide and biodiversity record of organisms observed in the Japan, Ryukyu and Izu-Ogasawara trenches in the Northwest Pacific Ocean between 4534 m and 9775 m. The guide compiles morphotaxa identifications, reference imagery and observational notes from approximately 460 hours of video footage obtained through baited lander and submersible transect surveys. A total of 108 morphotaxa were observed, which are listed here with locality and depth range. The illustrated occurrence list serves as a practical reference to aid in the identification of organisms in future image-based biodiversity assessments at these depths, to support voucher specimen collections and to provide a baseline record of faunal occurrence and distribution at these localities. Additionally, this study highlights the value of utilising multiple observation platforms to improve comprehensive biodiversity and behavioural assessments in abyssal and hadal ecosystems.

## Introduction

Sampling methodologies in the deepest 50% of the oceans have, in recent decades, seen a reversal in trends (Jamieson 2018). The first campaigns at abyssal (3000-6000 m) and hadal (> 6000 m) depths favoured the use of trawls, dredges and grabs (Wolff 1960, Belyaev 1989) over *in-situ* photography that was still in its infancy at the time (Heezen and Hollister 1971, Lemche et al. 1976). Although towed gear such as trawls are still used (e.g. Elsner et al. (2015), Shimanaga and Yanagi (2016)), contemporary exploration of these depths increasingly depends on *in-situ* imaging of marine geology and biodiversity (Jamieson 2018), which is non-destructive and can also be used in areas dominated by hard or complex substrate.

Amongst these, free-fall lander vehicles carrying baited cameras (stills and video) are the most frequently deployed tools at abyssal and hadal depths (> 4500 m), generating large datasets of mobile species attracted to bait (e.g. Jamieson et al. (2021), Swanborn et al. (2025b)). While most non-bait-attending species are observed serendipitously at baited cameras (e.g. Jamieson et al. (2011), Jamieson and Linley (2021)), crewed submersibles have recently expanded the observational capacity of taxa *in-situ* and also allow traversing of multiple habitat types (Jamieson et al. 2020, Jamieson and Vecchione 2022, Jamieson et al. 2022, Swanborn et al. 2025a).

Effective and sustainable management of deep-ocean ecosystems fundamentally relies on understanding the distribution of biodiversity. Knowledge of the diversity and distribution of species in the deepest 50% of the ocean lags greatly behind all other shallower regions (Webb et al. 2010). To prevent further widening of the gap between our understanding of shallow and deep biodiversity, deep-ocean imaging datasets are important in multiple ways: 1) they provide morphological information on species that may otherwise be lost due to sampling damage or preservation (e.g. Robison et al. (2017)); 2) they provide information on behaviour, species interactions and associations that are not always obvious in physical sampling (e.g. Aguzzi et al. (2012)); 3) they provide information on habitat preference and interactions with substrate and water column (e.g. Buhl‐Mortensen et al. (2010)) and 4) they can provide information with which to develop hypotheses and sampling design for future studies.

However, in-situ imaging at lower abyssal and hadal depths is complicated by several factors. There are rarely physical samples for morphological or molecular taxonomy to develop taxonomic keys to corroborate all identifications (Howell et al. 2019) and, with increasing depth, there is likely a disproportionately higher number of species that have never been collected. In addition, image quality and orientation may only provide limited information on diagnostic features required for identification, which may be further obscured by environmental factors as turbidity. Substantial volumes of image data may be generated, requiring significant computing power and the development of extensive and consistent databases and extracting quantitative, consistent information may be compromised by observer bias (Howell et al. 2014). Combined, these factors result in species identifications with the highest confidence still residing in higher taxonomic ranks (Horton et al. 2021).

The ideal approach for assessing biodiversity in lower abyssal and hadal ecosystems would be to undertake in-situ imaging and physical sampling concurrently (Howell et al. 2014, Rogers et al. 2022). This would enable taxonomic cataloguing, species descriptions and, more recently, associated molecular taxonomy and phylogenetics. However, until physical sampling in a variety of habitats at these depths becomes more commonplace, the reliance on image-based data for identifications will continue to be high. Raw image data and annotations are often lost or limited in the publication of more ecological scientific texts and, therefore, a wealth of highly useful reference material for future identifications can remain obscure. The lack of comprehensive species lists supported by visual reference material or field guides poses a challenge for many deep-sea imagery-based studies, especially so at hadal depths. The development of species lists or field guides, in addition to other scientific texts, is therefore essential for supporting future consistent taxon identification from still images or video (e.g. Amon et al. (2017), Simon-Lledó et al. (2023)).

This study contributes to that need by providing an imagery-based guide to the lower abyssal and hadal megafauna of the Japan, Ryukyu and Izu-Ogasawara trenches (including the Boso Triple Junction) between depths of 4534 and 9775 m. Crewed submersible observations in these trenches are limited as the Japanese national deep-sea submersible Shinkai 6500 is constrained to depths of 6500 m or less (Komuku et al. 2007) and the last full ocean depth rated submersible to visit these trenches was the French bathyscaphe *Archimède* in the 1960s (Bellaiche 1980, Komatsu and Ceccaldi 2024). The use of remotely-operated vehicles has been limited to the *Kaiko* ROV (Okutani and Fujiwara 2005) which was lost in 2003 (Buhl‐Mortensen et al. 2010, Anonymous 2004). Recent advances in full-ocean depth technology, including the platforms used in this study, have enabled biological observations at these depths, allowing new image-based records to be collected despite the historical scarcity of such data. Several observations from these trenches have been reported (e.g. Lindsay (2005), Lindsay and Miyake (2007), Fujii et al. (2010), Jamieson et al. (2012b), Jamieson et al. (2012a), Jamieson et al. (2023b), Jamieson et al. (2023a)) and some trawl records exist at hadal depths, but are limited in numbers and depth range (Horikoshi et al. 1990).

The following imagery-based guide contains in-situ observations and identifications of organisms encountered during baited lander deployments and crewed submersible transects at lower abyssal and hadal depths in the Japan, Ryukyu and Izu-Ogasawara trenches. Its purposes are to serve as a reference for future imagery-based surveys, to help provide information for the design of future sampling campaigns and to provide a baseline record of faunal occurrence in the lower abyssal and hadal zones of the Japan, Ryukyu and Izu-Ogasawara trenches. Ecological analyses of the baited landers dataset are discussed in Swanborn et al. (2025b) and of the submersible dives in Swanborn et al. (2025a).

## Materials and Methods

### Study Sites

**Figure 1.**
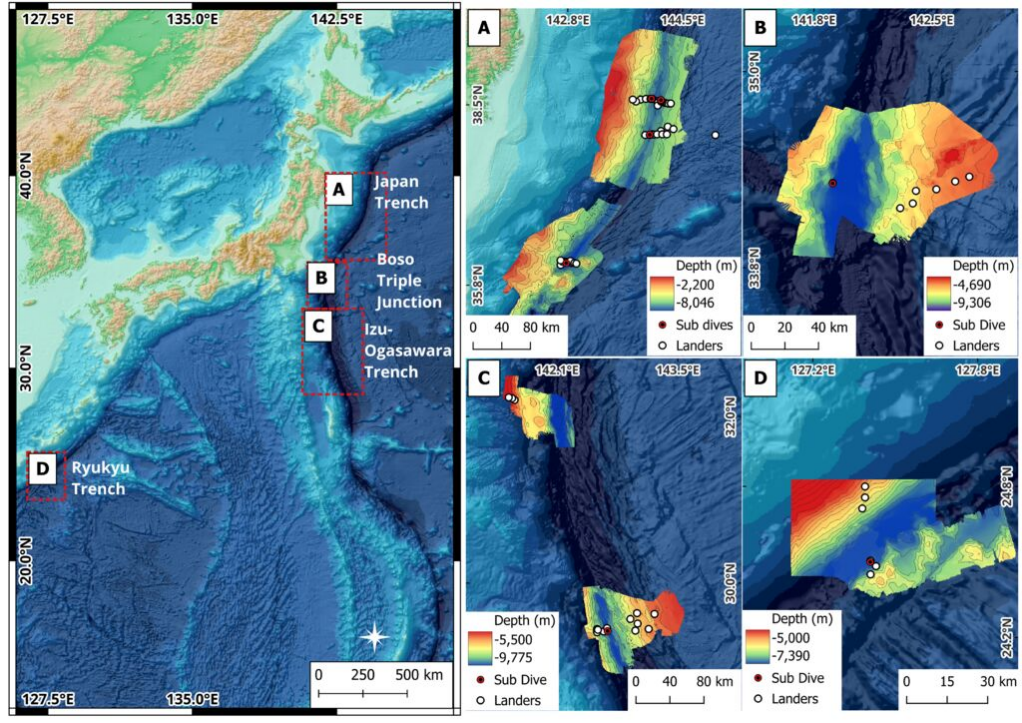
Bathymetry of the Northwest Pacific Ocean with specific study areas labelled. Inset maps relate to individual study sites described here in: Japan Trench (A), Boso triple junction (B), Izu-Ogasawara Trench (C) and Ryukyu Trench (D), with locations of submersible dives and lander deployments indicated. All regional elevation data sourced from the General Bathymetric Chart of the Oceans (GEBCO Compilation Group 2024) and multibeam bathymetry data at study sites acquired for this study onboard DSSV Pressure Drop, which was supplemented with GEBCO data for the Ryukyu Trench (D).

The Japan, Izu-Ogasawara and Ryukyu trenches (Fig. 1), all located within the Japanese exclusive economic zone (EEZ), were surveyed during the ‘Ring of Fire Expedition’ in August and September 2022 on DSSV *Pressure Drop*.

**Japan Trench**

Located parallel to the east coast of the Japanese mainland of Honshu, is the Japan Trench (Fig. 1A). It extends 611 km and covers an area of 37,854 km² beyond 6000 m depth. The Japan Trench is formed as the Pacific Plate subducts beneath the Okhotsk (minor) plate at a convergence rate of approximately 9 cm/yr (Nishikawa et al. 2023). The deepest point is 8001 m at 36.0872° N, 142.7272° E (this study). In the north, a subducting seamount partitions it from the Kuril-Kamchatka Trench and, in the south, the westernmost seamount of the Joban Seamount Chain separates it from the Izu-Ogasawara Trench (Yamazaki and Okamura 1989). The Japan Trench is seismically active and known for tsunamigenic megathrust earthquakes (Ueda et al. 2023) that result in large mass wasting events at depth, resulting in large volumes of re-suspended sediments (Itou et al. 2000, Oguri et al. 2013, Kioka et al. 2019).

**Izu-Ogasawara Trench and Boso triple junction**

The Izu-Ogasawara trench (or Izu-Bonin Trench; Plank et al. (2007)) connects to the Japan Trench at the intersection of the Joban Seamount Chain in the vicinity of the Boso triple junction (trench-trench-trench or TTT type; Ogawa and Yanagisawa (2011)) (Fig. 1C). The Izu-Ogasawara Trench is formed as the pacific Plate is subducted beneath the Philippine Plate at a convergence rate of approximately 3-6 cm/yr (Stern et al. 2003). The triple junction is where the Pacific, Eurasian and Philippine tectonic plates converge (Soh et al. 1988) (Fig. 1B). The trench extends between the Japan Trench and the Izu-Ogasawara Plateau for 1122 km and hosts 105,328 km^2^ of seafloor > 6000 m. It has a maximum depth of 9775 m at 29.4262° N, 142.7075° E (this study). The northern part of the trench subduction occurs largely by aseismic slip, resulting in no great earthquakes (Fukao et al. 2021). In contrast, the southern sector of the trench is characterised as a double seismic zone (DSZ) (Nakata et al. 2019).

**Ryukyu Trench**

Parallel to the Japanese Nansei-Shotō Islands between Taiwan and Kyūshū is the Ryukyu Trench (or Nansei-Shotō Trench; Nishizawa et al. (2017)) (Fig. 1D). This trench is 1012 km long and covers a surface area of 39,073 km^2^ > 6000 m. The deepest point is 7339 m at the south-western end of an elongated depression at 24.4752° N, 127.4088° E (this study). The trench is formed as the Philippine Plate is obliquely subducted beneath the continental crust of the Eurasian Plate at a rate of approximately 52 mm per year (Nishizawa et al. 2009). The trench exhibits very low-frequency earthquakes (VLFEs; Ando et al. (2012)) and is known to produce frequent slow slip events (SSEs) that can last for ~ 1 month (Heki and Kataoka 2008).

### Equipment and operations

Survey areas were first mapped using a hull-mounted EM124 multibeam echosounder (MBES; Kongsberg, Norway; Bongiovanni et al. (2022)). In-situ observations were made using three identical free-fall landers (Skaff, Flere and Closp) equipped with high-definition video cameras (IP Multi SeaCam 3105; Deep Sea Power and Light, San Diego, CA) and conductivity, temperature and depth (CTD) probes (SBE 49 FastCAT, SeaBird Electronics, Bellevue, WA). Additionally, video transects were conducted using the full ocean depth submersible *Limiting Factor*, with footage recorded via two forward- and downward-facing cameras. Depth was recorded using two CTD probes. Cameras and CTD probes on the submersible were the same as on the landers. Full details of lander and submersible deployments are provided in Table 1 and Suppl. material 1, and information on landers and the submersible *Limiting Factor* can be found in Jamieson et al. (2019).

In the Japan Trench (Fig. 1A), four submersible dives were undertaken, one at its deepest point (8002 m, 36.087°N, 142.727°E) and three around the location of the 2011 Tōhoku earthquake epicentre. Of these, one was located on the trench axis (7529 m, 38.045°N, 143.999°E), one on the adjacent forearc (7324 m, 38.594°N, 144.026°E) and one on the adjacent downriding slope (7381 m, 38.571°N, 144.169°E). Thirty-two successful lander deployments were conducted in the Japan Trench, of which six were around its deepest point and the remaining 26 were around the location of the Tōhoku earthquake epicentre (Ueda et al. 2023) (Fig. 1A).

In the Izu-Ogasawara Trench (Fig. 1B and C), two submersible dives were undertaken, one in the southern end at the deepest point (9775 m, 29.426°N, 142.708°E) and one in the north at the base of the Boso triple junction (9137 m, 34.288°N, 141.870°E). A total of 21 successful lander deployments were also conducted along the length of the Izu-Ogasawara Trench, of which seven were located in the vicinity of the Boso triple junction (34°N), three at 32°N and the remaining 11 at 29°N (Fig. 1B and C).

In the Ryukyu Trench (Fig. 1D), one submersible dive was undertaken to its deepest point (7322 m, 29.475°N, 127.409°E). Six lander deployments were conducted on the overriding plate, of which three were located around the deepest point of the trench and three on the landward slope (Fig. 1D).

### Video annotation and data analysis

Video footage recorded *in-situ* from the baited landers and submersible dives was continuously annotated using EventMeasure (SeaGIS Pty Ltd, version 6.42) to generate records of taxa present. Submersible dive footage was only annotated if the submersible was on-bottom. Lander video footage was only annotated when the lander was on the seafloor (bottom time) using MaxN (also known as N_max_) which is a conservative metric of relative abundance and refers to the maximum number of individuals of each taxon observed at a single point in time (Priede et al. 1994).

All visible mobile fauna were identified to the lowest possible taxonomic rank using morphological characteristics. All identifications were derived from review of video footage from which still images were extracted, allowing us to distinguish morphotaxa using a combination of morphology, movement and behaviour. Where species-level identifications were not possible, taxa were placed in putative ‘morphotaxa’. The level of taxonomic precision achieved was indicated using the open taxonomic nomenclature signs recommended for image-based identifications by Horton et al. (2021). A suffix was added to specify the taxonomic rank (e.g., “fam.”, “gen.”, “sp.”). The designation “indet” was used when a single observed form could not be reliably identified beyond a given taxonomic level because diagnostic characteristics were unresolvable from the imagery. The designation “spp.” was used when multiple visual forms were observed within a given taxonomic rank, but lacked sufficient detail to be consistently separated across all annotated imagery, and therefore grouped (e.g. “Mysidae spp.”). Representative reference images were extracted for each morphotaxon; however, images of very poor quality were excluded from the ID guide and marked as ‘ns’ (not shown).

Submersible and lander datasets were combined to provide an overview of morphotaxon occurrences across trenches (Table 2, Suppl. material 2). Depth ranges for each observed morphotaxon were determined, based on the minimum and maximum depths of observations.

## Results

In total, ~ 425 h of lander video footage and ~ 35.5 h of submersible video footage was collected at the seafloor.

The total number of identified morphotaxa in the trenches was 78, of which 45 (57.6%) were in the Izu-Ogasawara Trench, 61 (78.2%) in the Japan Trench and 27 (34.6%) in the Ryukyu Trench. In addition, 30 groups with indeterminable morphotaxa numbers (‘spp’) were also included. These included groups of small individuals that swarm (e.g. Amphipoda and Mysida), groups with small indeterminable body sizes (e.g., Annelida, munnopsid Isopoda, Ophiuroidea, elasipodid Holothuroidea), individuals of groups that were often drifting too far from the camera to confidently identify (e.g., Appendicularia, Narcomedusae, Trachymedusae and Ctenophora) and other groups that are notoriously difficult to identify from images (e.g., Monothalamea). Of these 30 groups, 23 (76.6%) were in the Izu-Ogasawara Trench, 28 (93.3%) in the Japan Trench and 21 (70%) in the Ryukyu Trench. Sixteen groups (53.3%) were found in all three trenches, 10 (33.3%) in two trenches and 4 (13.3%) in a single trench. Assuming the latter 30 groups represent at least one morphotaxa each, there were a total of at least 108 across all the trenches. Of these, 34 (31.5%) were found in all three trenches, 28 (25.9%) in two of the trenches and 46 (42.6%) in just one trench. Note that the number of submersible dives and lander deployments varied across trenches, meaning these values are indicative rather than strictly representative assessment of each trench.

### Annelida

**Figure 2.**
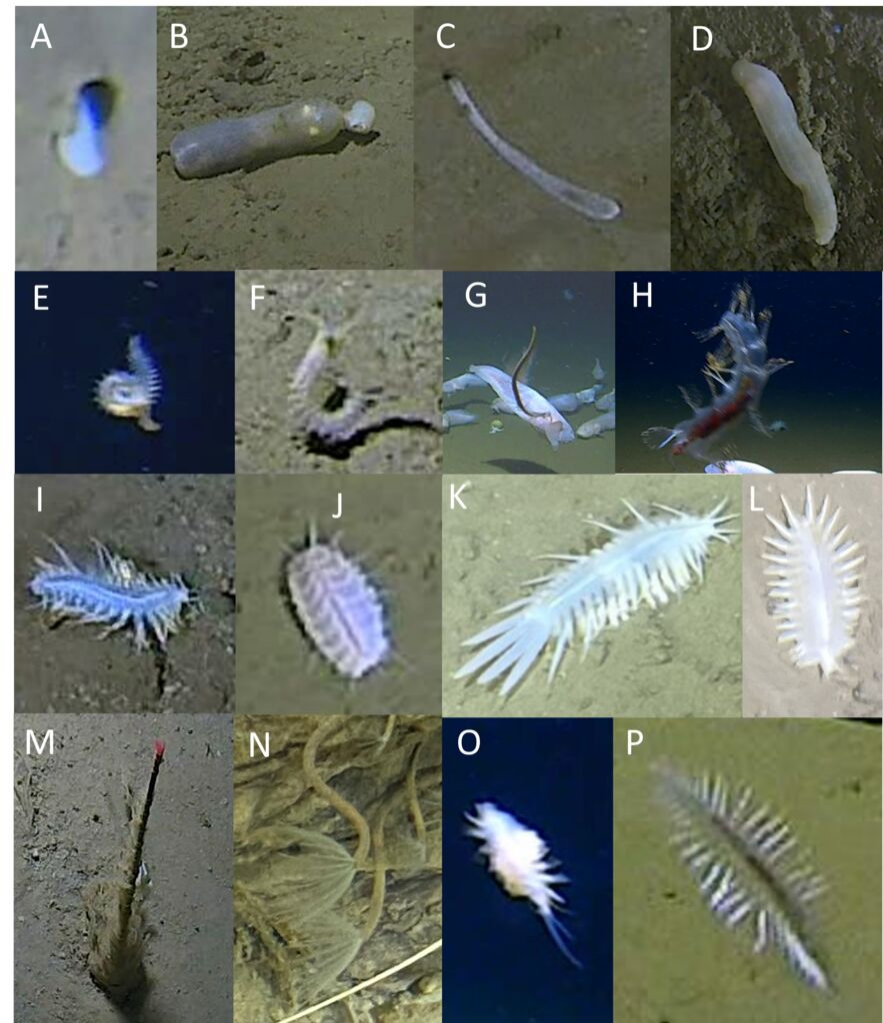
Annelida. **A** Echiuroidea fam. indet. 1; **B** Echiuroidea fam. indet.; 2 **C** Echiuroidea fam. indet. 3; **D** Echiuroidea fam. indet. 6; **E** Annelida indet. 1; **F** Annelida indet. 2; **G** Annelida indet. 3; **H** Flabelligeridae spp.; **I** Macellicephalinae spp.; **J** Lepidonotopodini spp.; **K** Macellicephalinae gen. indet. 1; **L** Macellicephalinae gen. indet. 2.; **M** Sabellidae spp.; **N** Sabellidae gen. indet. 1; **O** Annelida spp.; **P** Acrocirridae spp.

Of the 18 morphotaxa identified in the Annelida phylum, 15 showed morphological features that allowed identification beyond phylum level; however, three morphotaxa could not be assigned confidently beyond Annelida indet. (Fig. 2E-G). Of these, Annelida cls. indet. 1 and Annelida cls. indet. 2 (Fig. 2E and 2F) appeared morphologically similar however, their swimming styles differed. Annelida indet. 1 exhibited torsional rotations of the body axis while indet. 2 moved with lateral flexion from side-to-side. The other Annelida were represented by four identifiable orders (Echiuroidea; Fig. 2A-D, Phyllodocida; Fig. 2I-L, Sabellida; Fig. 2M, N and Terebellida; Fig. 2H-P). Six morphotaxa were identified in the Echiuroidea order (with an additional Echiuroidea spp. comprising small indeterminable morphospecies), four in the Phyllodocida order (subfamilies Macellicephalinae and Lepidonotopodini) and two in the Terebellida (families Acrocirridae and Flabelligeridae) order, the others being two morphotaxa in the Sabellidae order. None of the annelid morphotaxa were determined to the species level and, whilst observations of annelids were abundant, their small size complicated confident assignment of morphotaxa based on morphology. For this reason, several annelid morphotaxa identifications relied on movement and behaviour. Apart from two, all annelids observed were present in the Japan Trench, while seven were observed in the Ryukyu Trench and six in the Izu-Ogasawara Trench. All were observed at hadal depths. All were benthic taxa, except for polynoids in the subfamily Macellicephalinae, which included Flabelligeridae (possibly including the genus *Flota*; Fig. 2H) and three unidentified morphotaxa of annelids that were observed swimming off the bottom (Fig. 2E-G). Of the three echiuroids, Echiuroidea fam. indet. 1 (Fig. 2A) and Echiuroidea fam. indet. 3 (Fig. 2C) were frequently observed appearing from burrows; however, the size of their burrow and proboscis shape were consistently different from one another. Echiuroidea fam. indet. 2 (Fig. 2B) resembled Urechidae, but family-level identification could not be confirmed with certainty. All Annelida were mobile, except for the two Sabellidae morphotaxa which were observed in a ridged or semi-ridged tube protruding vertically from the seafloor. Their slow mobility or sessile nature meant that the majority were observed by submersible rather than from the lander.

Historical records show two unknown species of polynoid, *Macellicephala* “sp. A” and “sp. B” from 7420-7450 m on the axis and 6700-7400 m on the adjacent slope of the Japan Trench (Horikoshi et al. 1990). The genus *Flota* has previously been reported at a maximum depth of 6444 m in the Japan Trench (Lindsay 2005).

### Arthropoda

**Figure 3.**
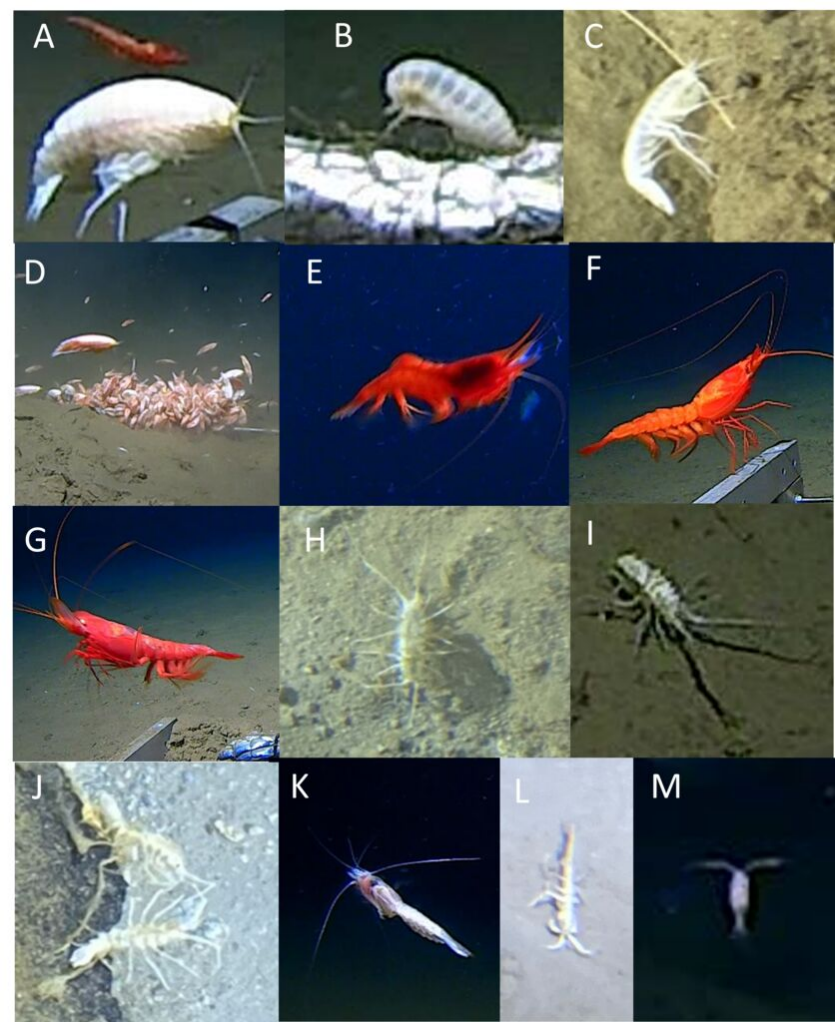
Arthropoda (Malacostraca). **A** *Alicella gigantea*; **B** *Eurythenes* spp.; **C** *Princaxelia jamiesoni*; **D** Amphipoda spp.; **E** *Heterogenys* sp. indet. 1; **F** *Cerataspis monstrosus*; **G** *Benthesicymus crenatus*; **H** Munnopsidae spp.; **I** *Rectisura herculea*; **J** Antarcturidae gen. indet.; **K** Mysidae spp.; **L** *Gigantapseudes* sp.; indet.; **M** Copepoda spp.

The Arthropoda were represented by two classes (Malacostraca and Copepoda), five orders, ten families (plus two unknown) forming 13 morphotaxa. Within the Malacostraca, five orders were observed: Amphipoda, Decapoda, Isopoda, Mysida and Tanaidacea (Fig. 3, Table 2).

In the Amphipoda order, multiple species were present within the Alicelloidea and Lysianassoidea superfamilies. Due the typically small body size of abyssal and hadal amphipods and their propensity to aggregate at baited lander in high numbers complicating identifications as well as counts, these amphipods were collectively listed here as Amphipoda spp. Known species within these trenches and these depths are *Hirondellea gigas* (Birstein and Vinogradov, 1955), *Paralicella microps* (Birstein and Vinogradov, 1958), *Eurythenes gryllus* (Lichtenstein in Mandt, 1822) and *Bathycallisoma schellenbergi* (Birstein and Vinogradov, 1958), although there are no records from the Ryukyu Trench (Jamieson and Weston 2023). Due to their abnormally large body size, the ‘supergiant amphipod’ *Alicella gigantea* Chevreux 1899 (Fig. 3A), the ‘giant amphipod’ *Eurythene*s sp. indet. (Fig. 3B) and the predatory amphipod *Princaxelia jamiesoni* Lörz 2010 (Fig. 3C) were identified from both landers and submersible footage. *A. gigantea* was observed in all three trenches for the first time (total depth range = 6529-8022 m), around their known depth range (6253-8903 m; Jamieson and Weston (2023)). *A. gigantea* was most conspicuous in baited lander footage, whereby they approach the bait by swimming close to the bottom and ultimately consuming the bait. *Eurythene*s sp. indet. was absent in the Izu-Ogasawara Trench between 6941 m and 7310 m, close to the depth Belyaev (1989) reported *E. gryllus*, which is likely *sensu lato* (Jamieson and Weston 2023). Their behaviour was akin to that of *A. gigantea*. *Princaxelia jamiesoni* was absent from Ryukyu Trench which is in accordance with collections and observation of Lörz (2010) and Jamieson et al. (2012b), respectively. They were observed in baited camera footage preying upon smaller scavenging amphipods (as per Jamieson et al. (2012b); however, in submersible footage, they were often seen stationary on rocks or hard substrate.

The Decapoda were dominated by two species; *Cerataspis monstrosus* Gray 1828 (Aristeidae) between 4534 and 6692 m and *Benthesicymus crenatus* Spence Bate, 1881 (Benthesicymidae) between 4534 m and 7571 m (Fig. 3F and G). The former is a scavenging species, while the latter preys upon scavenging amphipods and both are most readily recorded using baited cameras. These types of feeding behaviour and depth ranges are typical for these two species. Although they were only known from the Japan Trench (Swan et al. 2021), they were observed in all three in this study. *Heterogenys* sp. indet. 1 of the Acanthephyridae family was also observed between 4534 m and 6692 m in all trenches (Fig. 3E). A fourth morphotaxon was observed at 5941 m in the Ryukyu Trench, but was too small for species-level identification.

One family-level grouping (Munnopsidae spp.) and one known species of isopod (*Rectisura herculea*, Birstein 1957) were observed (Fig. 3H and I). Munnopsidae are a cryptic family and difficult to delineate to lower taxonomic levels from imagery, but were recorded between 4534 m and 9734 m across all trenches. *Rectisura herculea* (Birstein, 1957) were present in the Japan and Izu-Ogasawara trenches between 5525m and 8291 m. These epibenthic species have been recorded in these trenches at similar depths before (seeHorikoshi et al. 1990; note, the genus at the time was *Storynthgura*; and Jamieson et al. 2012a). *Rectisura herculea* was quite common and conspicuous in the Japan and Izu-Ogasawara trenches; therefore, its absence from the Ryukyu Trench is likely real.

Valviferan isopods (Antarcturidae gen. indet.) were seen between 7165 m and 7345 m in the Japan Trench (Fig. 3J). They were seen perched along the edges of rocks protruding from the seafloor with their pereiopods elevated in the current.

Mysids (Mysidae spp.) were observed at all depths across all trenches (Fig. 3K). They typically swim close to, but above the seafloor, often in swarms of scavenging amphipods around bait or resting on the seafloor in submersible transects. Usually, they are either white or red, but their small size made it difficult to determine if body colour indicates two different morphotaxa.

In the Japan and Ryukyu trenches, the giant tanaid *Gigantapseudes* sp. indet. was seen between 6937 m and 7345 m in submersible videos (Fig. 3L). They were visible on the seafloor, often nearby a burrow as Kakui and Fujiwara (2020) reported from the Ryukyu Trench.

Copepods of unknown order(s) were seen between 4913 m and 7621 m in the Japan and Izu-Ogasawara trenches (Fig. 3M) which is a depth range similar to that of other copepods found in neighbouring trenches (Markhaseva and Renz 2025).

### Chordata

**Figure 4.**
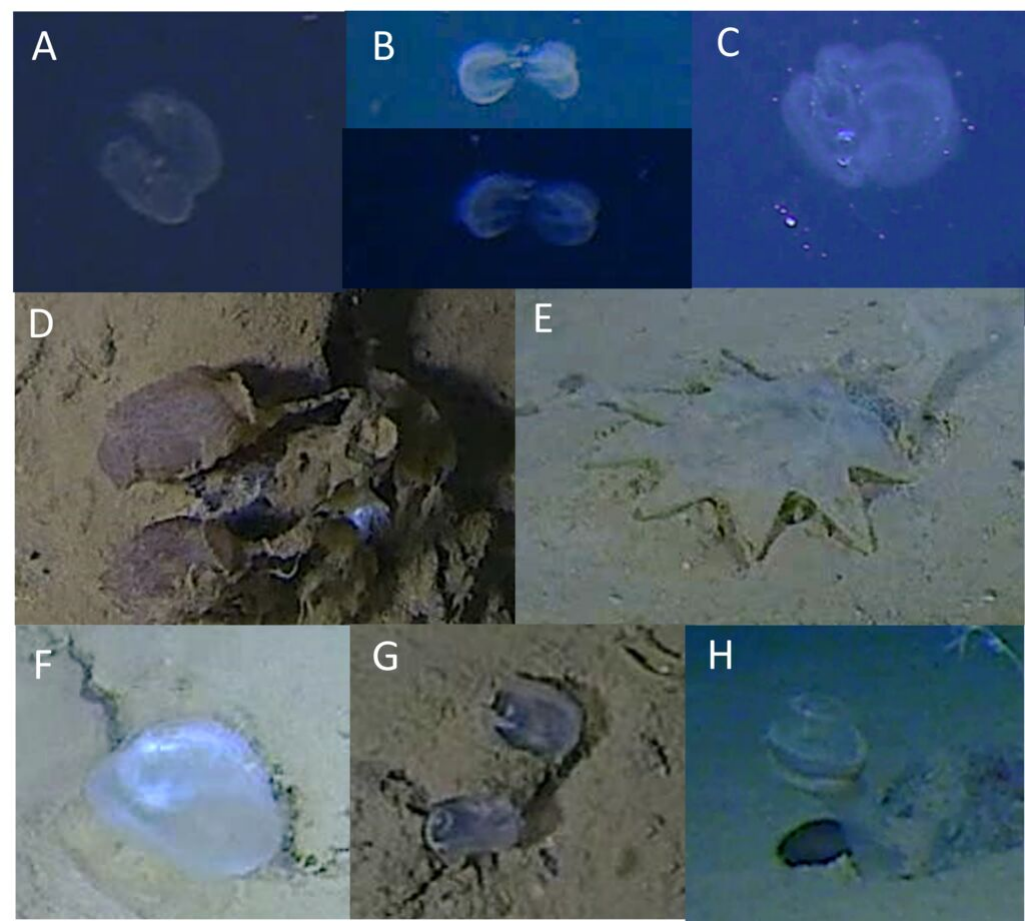
Chordata (Appendicularia and Ascidiacea). **A** Copelata spp.; **B** Oikopleuridae spp.; **C** Fritillariidae spp.; **D** Ascidiacea spp.; **E** *Octacnemus* sp. indet.; **F** Octacnemidae gen. indet. 1; **G** Octacnemidae gen. indet. 2; **H** Octacnemidae gen. indet. 3.

**Figure 5.**
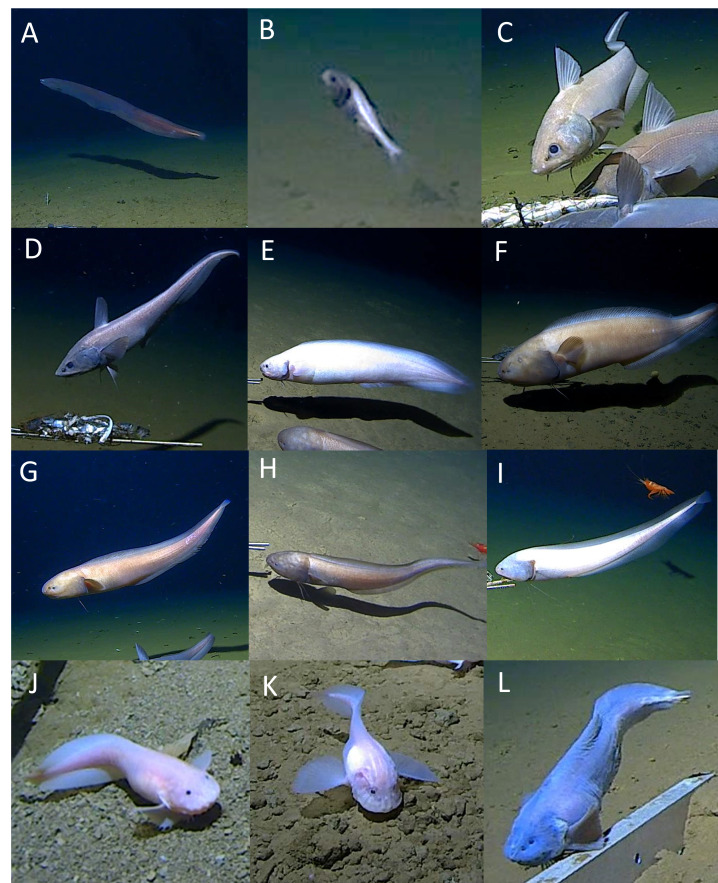
Chordata (Teleostei). **A** *Ilyophis* sp. indet.; **B** *Abyssoberyx* sp. indet.; **C** *Coryphaenoides armatus*; **D** *Coryphaenoides yaquinae*; **E** *Barathrites iris*; **F** *Bassozetus* sp. indet. 1; **G** *Bassozetus* sp. indet. 2; **H** *Bassozetus* sp. indet. 3; **I** *Bassozetus* sp. indet. 6; **J** *Pseudoliparis* sp. indet. 1; **K** *Pseudoliparis* sp. indet. 2; **L** *Pseudoliparis* sp. indet. 3.

The Chordata were represented by three classes: Appendicularia, Ascidiacea and Teleostei (Figs 4, 5, Table 2). The Appendicularia were represented by three morphotaxa in the Copelata order: Fritillariidae spp. (5042-6579 m Japan and Izu-Ogasawara trenches; Fig. 4C), Oikopleuridae spp. (4534-5941 m, Izu-Ogasawara and Ryukyu trenches; Fig. 4B) and Copelata spp. (4534-8336 m, all trenches, Fig. 4A). All were observed from baited landers, drifting past the camera in the near-bottom currents. If the housing was ovoid, showed two distinct chambers and was centrally positioned, an individual was classified as Oikopleuridae; if it was elongate and bilaterally symmetrical, it was identified as Frittilariidae (Alldredge 1976, Bone 1998); otherwise, they were left at the order level. Five Ascidiacea morphotaxa were observed. One was too cryptic to determine individual morphotaxa (Ascidiacea spp.; 7096-7345 m; Japan Trench; Fig. 4D) and one was of an unknown species (*Octacnemus* sp. indet.; 6606-7344 m; Izu-Ogasawara and Japan trenches; Fig. 4E) (Mandre and Rouse 2025). The other three were of the Octacnemidae (Phlebobranchia) family (Mandre and Rouse 2025); Octacnemidae gen. indet. 1 (7421-7290 m; Japan Trench; Fig. 4F), Octacnemidae gen. indet. 2 (7170-8077 m; Fig. 4G) and Octacnemidae gen. indet. 3 (7345-8077 m; Izu-Ogasawara and Japan trenches; Fig. 4H). No Ascidians were observed in the Ryukyu Trench.

The bony fish (Teleostei) were represented by five orders and five families. Twelve morphospecies or species were identified following Jamieson et al. (2021). The synaphobranchid eel *Ilyophis* sp. indet. (Synaphobranchidae) was seen at 4534 m in the Izu-Ogasawara Trench (Fig. 5A). The pricklefish *Abyssoberyx* sp. indet. (Stephanoberycidae) was seen at 5459 m in the Izu-Ogasawara Trench (Fig. 5B). The grenadier *Coryphaenoides armatus* (Hector, 1875) (Macrouridae) was seen in the Izu-Ogasawara Trench between 5042 m and 5459 m (Fig. 5C). Another macrourid, *C. yaquinae* (Iwamoto & Stein, 1974), was seen in the Japan and Izu-Ogasawara trenches between 4534 m and 7259 m (Fig. 5D). The deepest observation of *C. yaquinae* represents the deepest known use of a gas-filled swimbladder in fishes (Priede et al. 2024). Five morphospecies of cusk eel (Ophidiidae) were seen, with *Barathrites iris* Zugmayer, 1911 observed at 5110-5941 m in the Ryukyu Trench (Fig. 5E). The other four were morphospecies of the genus *Bassozetus*, labelled here as *Bassozetus* sp. indet. 1, 2, 3 and 6 (Fig. 5F-I) in keeping with the categorisation of Jamieson et al. (2021). Three morphospecies of snailfish (Liparidae) were seen: *Pseudoliparis* sp. indet. 1, 2 and 3 (Fig. 5J-L; corresponding to Liparid sp. indet. 1, 3 and 2 in Jamieson et al. (2021)). They were found in all trenches, except *Pseudoliparis* sp. indet. 2, which was absent from the Ryukyu Trench. Their depth ranges were 6023-8336 m, 6579-7621 m and 6860-8022 m, respectively. *Pseudoliparis* sp. indet. 1 at 8336 m in the Izu-Ogasawara Trench is the deepest recorded fish (Jamieson et al. 2023b). Another putative morphospecies of liparid is discussed further in the Discussion under the section ‘Rarities and Peculiarities’, but is not included in the species list.

All fish were observed by baited camera; however, the three *Pseudoliparis* and four *Bassozetus* morphospecies attended the bait to prey upon scavenging amphipods. *Barathrites iris, Ilyophis* sp. indet. and the two macrourids all scavenged upon the bait. *Abyssoberyx* sp. indet. was observed incidentally, irrespective of the bait.

### Cnidaria

**Figure 6.**
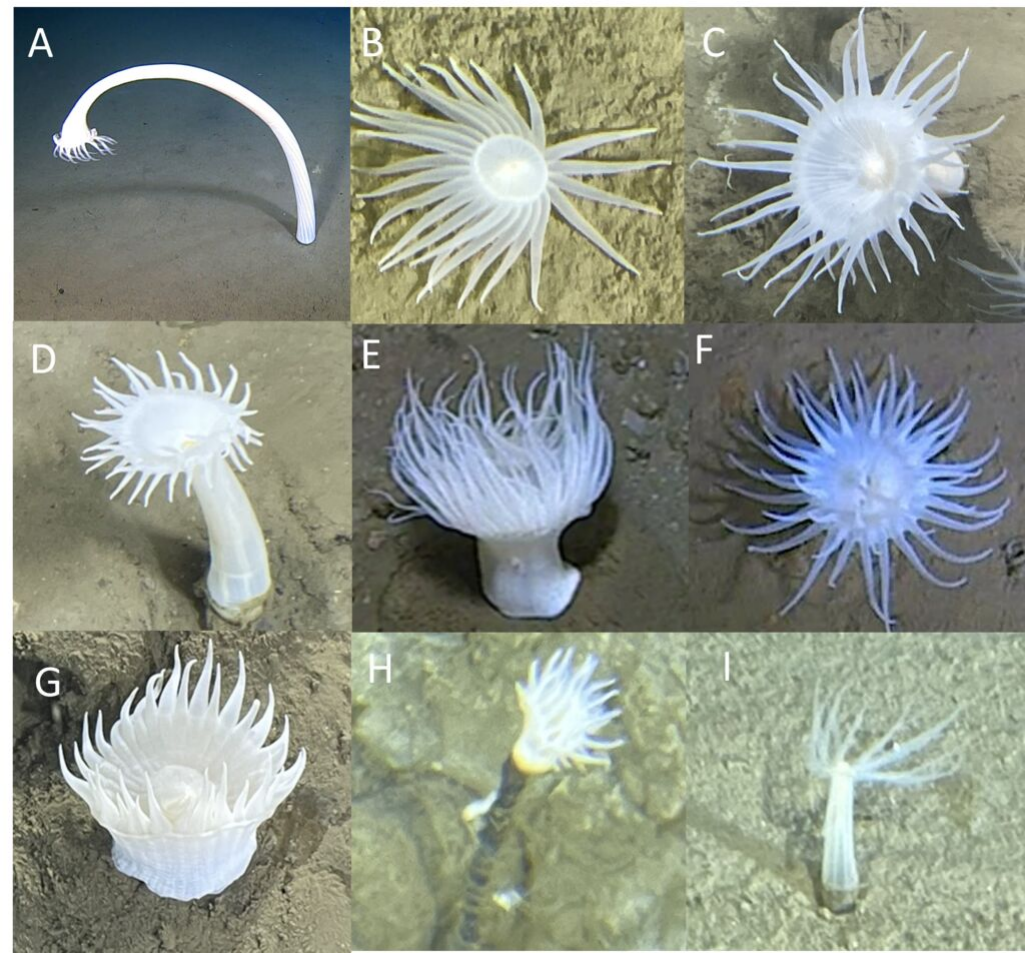
Cnidaria (Hexacorallia and Anthozoa). **A** Actiniaria fam. indet. 1; **B** Actiniaria fam. indet. 2; **C** Actiniaria fam. indet. 3; **D** Actiniaria fam. indet. 4; **E** Actiniaria fam. indet. 5; **F** Actiniaria fam. indet. 6; **G** Actiniaria fam. indet. 7; **H** *Galatheanthemum* sp. indet. 1.; **I** Anthozoa spp.

**Figure 7.**
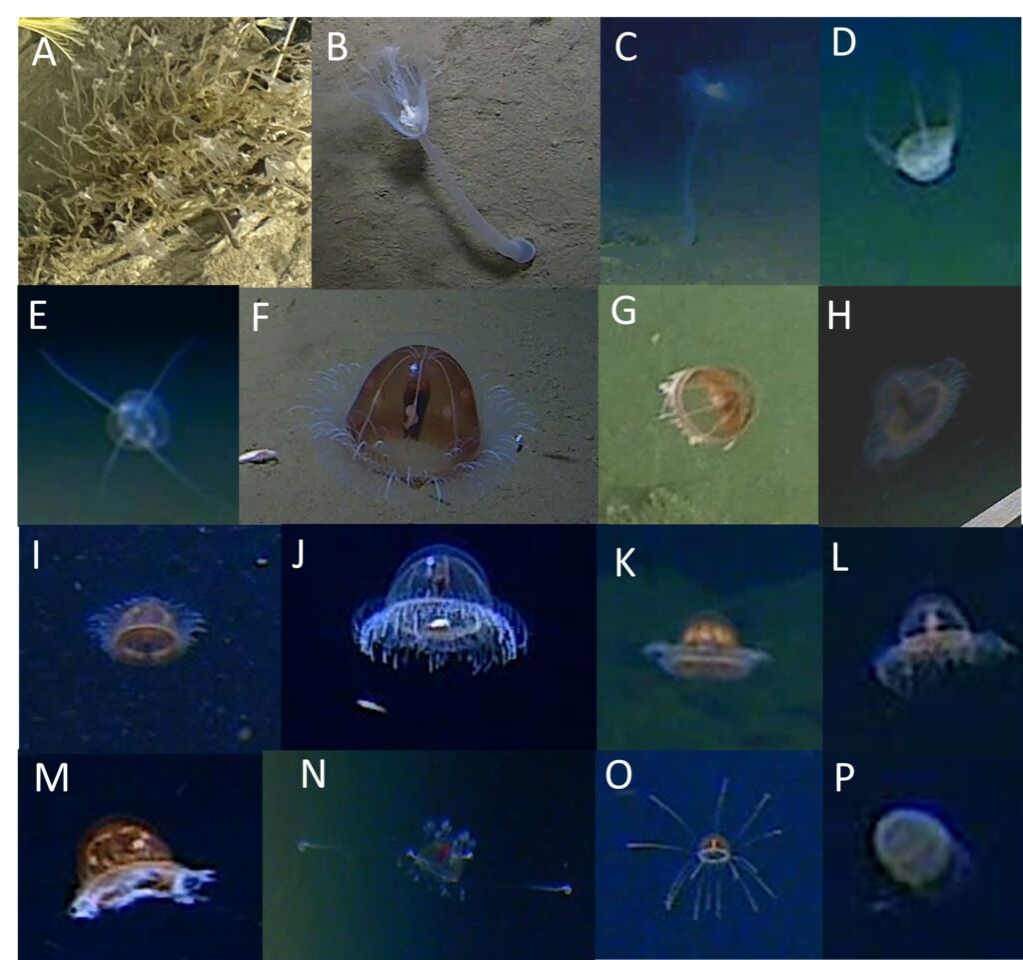
Cnidaria (Hydrozoa and Scyphozoa). **A** Hydrozoa ord. indet. 1; **B** *Branchiocerianthus* sp. indet.; **C** Corymorphidae gen. indet.; **D** Narcomedusae spp.; **E** Aeginidae spp.; **F, G** *Benthocodon* spp.; **H, M** Rhopalonematidae spp.; **N, O** *Crossota* spp. (C. aff. millsae); **P** Coronatae fam. indet.

The Cnidaria were represented by three classes: Hexacorallia, Hydrozoa and Scyphozoa (Figs 6, 7, Table 2).

Actiniarians (Actinaria spp.) were observed in all three trenches between 6485 m and 9617 m. They were often too small and cryptic to assign a specific morphotaxon. Nonetheless, seven actiniarian morphotaxa were identified, mainly in the Izu-Ogasawara and Japan trenches. Morphotaxa were distinguished through consistent differences in column length and width, column diameter to oral disc ratio, whorl count and tentacle number and morphology.

Actiniaria fam. indet. 1, 2, 3, 4 and 7 were all observed at similar depth ranges in the Izu-Ogasawara and Japan trenches (7528-9775 m, 7526-9137 m, 7095-9745 m, 7082-9734 m and 7168-9136 m, respectively; Fig. 6A-D, G). Actiniaria fam. indet. 1 (Fig. 6A) was distinguished by the presence of a very extended column, whereas a column was practically absent in Actiniaria fam. indet. 2 (Fig. 6B). Actiniaria fam. indet. 3 and 4 (Fig. 6CD) appeared to have similar basal disc morphology, but differed in an increased column length to oral disc ratio and tentacle count in Actiniaria fam. indet. 4. Actiniaria fam. indet. 7 (Fig. 6G) had the greatest column diameter to oral disc ratio of morphotypes observed. Actiniaria fam. indet. 5 and 6 were only seen in the Japan Trench at 7180-7363 m and 7527-7528 m, respectively (Fig. 6E and F). Compared to other morphotypes, Actiniaria fam. indet. 5 (Fig. 6E) has a high density of closely-spaced tentacles exceeding the length of the column. Actiniaria fam. indet. 6 (Fig. 6F) featured a tentacle arrangement in multiple whorls, extending into the oral disc.

Other morphotaxa observed in the Actiniaria included *Galatheanthemum* sp. indet. 1 of the Galatheanthemidae family, seen between 7300 m and 7500 m in the Japan Trench (Fig. 6H). Anthozoa spp. were observed on soft sediments in all trenches (7309-9745 m; Fig. 6I).

The Hydrozoa were represented by three known orders (Anthoathecata, Narcomedusae and Trachymedusae) and one indeterminable order. Ten hydrozoan morphotaxa were identified.

Within the Anthoathecata, there were two hydrozoan morphotaxa in the Corymorphidae family: the giant hydroid *Branchiocerianthus* sp. indet at 8022 m and Corymorphidae gen. indet. at 4913 m, both in the Japan Trench (Fig. 7B and C, respectively). Two morphotaxa of were observed in the Narcomedusae order, the first being small individuals grouped under Aeginidae spp. between 6825 m and 9773 m in the Izu-Ogasawara and Japan trenches and Narcomedusae spp. between 5110 m and 8000 m in the Japan and Ryukyu trenches (Fig. 7D and E).

The Trachymedusae *Benthocodon* spp. (Rhopalonematidae) were found at depths ranging from 7183 m to 9773 m in the Izu-Ogasawara and Japan trenches (Fig. 7F and G). Other Rhopalonematidae were observed between 5459 m and 9773 m in the Izu-Ogasawara and Japan trenches (Fig. 7H–M). However, due to poor image quality, it was not possible to clearly identify morphological features, such as centripetal canals stemming from the ring canal and gonad positions, which are key for distinguishing genera. The Rhopalonematidae spp. group includes species with a peduncle, such as the *Benthocodon* and *Pectis* genera (e.g. Fig. 7H, J and L), as well as other individuals where even the presence of a peduncle could not be ascertained. Between 5110 m and 6023 m in the Ryukyu and Izu-Ogasawara trenches, the Trachymedusa *Crossota* spp. were observed (Fig. 7N and O). In some cases, they resembled *C. millsae* Thuesen 2003, but exact species identities could not be confirmed in all instances.

A single scyphozoan of the Coronatae order (Periphyllidae or Paraphyllinidae) was seen at 5993 m in the Japan Trench (Fig. 7P).

### Ctenophora

**Figure 8.**
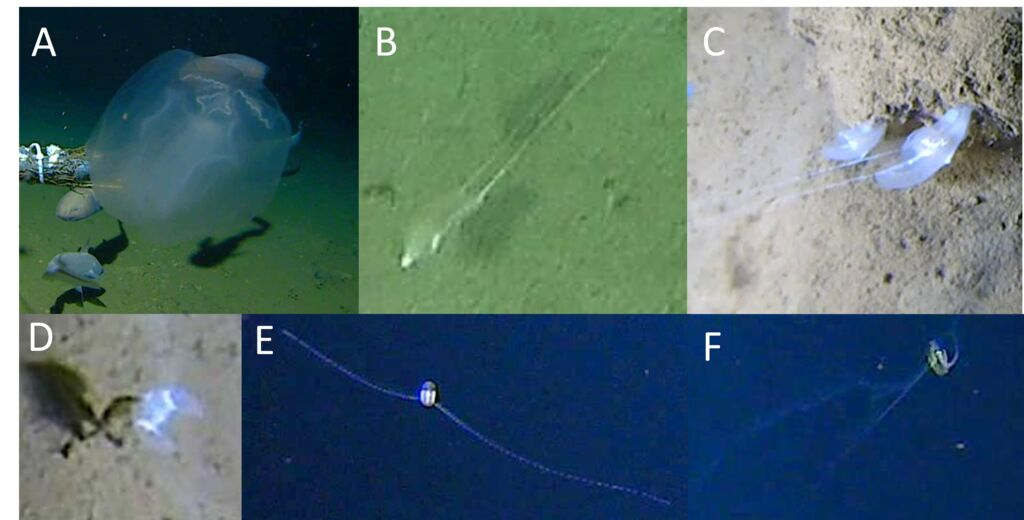
Ctenophora (Tentaculata). **A** Lobata fam. indet.; **B** Cydippida fam. indet. 1; **C, D** Lyroctenidae gen. indet.; **E, F** Cydippida spp. Ctenophora spp. are not shown due to low image quality.

Ctenophores were observed in the Tentaculata class, belonging to the orders Platyctenida, Cydippida and Lobata (Fig. 8, Table 2). Lobata fam. indet. were seen drifting past baited cameras close to the seafloor between 5042 m and 6177 m in all trenches (Fig. 8A). In the Cydippida order, video data supported Cydippida fam. indet. 1 (Fig. 8B) as the same morphotype as reported in the Ryukyu Trench by Lindsay and Miyake (2007). This study represents its first records in the Japan and Izu-Ogasawara trenches, thereby also extending its known depth range to 9136 m. Other Cydippids (Cydippida fam. indet., Fig. 8EF) were observed in all trenches at depths between 4534 m and 7519 m. The benthic ctenophore Lyroctenidae gen. indet. (Platyctenida, 7091-8001 m) was frequently seen attached to hard substrate in the Japan Trench (Fig. 8C and D). Additionally, smaller individuals were observed in the Japan Trench at 8000 m, but, as these could not be identified to lower taxonomic level, these were collectively classified as Ctenophora spp.

### Echinodermata

**Figure 9.**
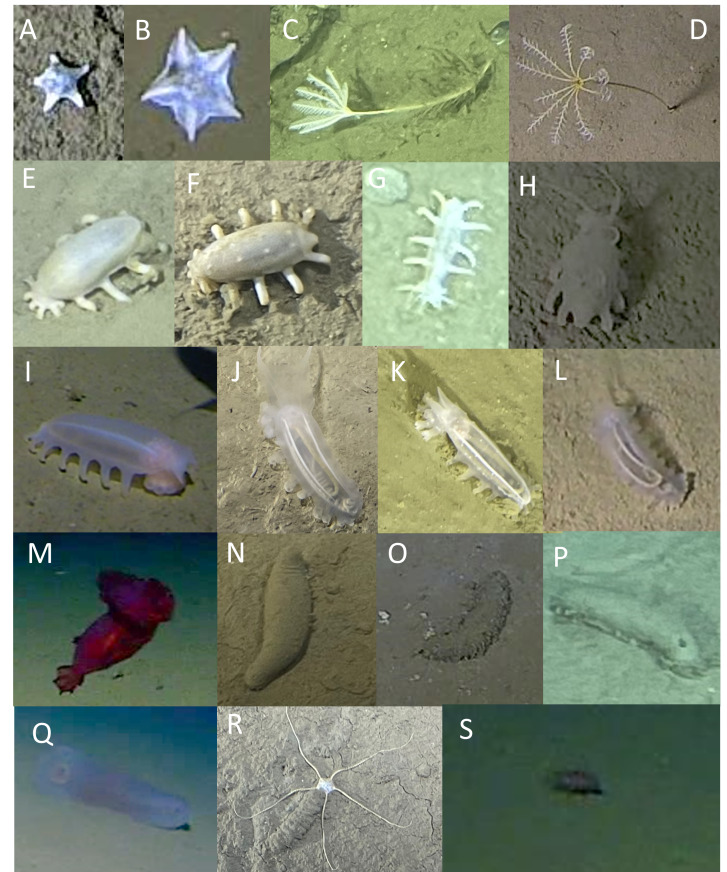
Echinodermata (Asteroidea, Crinoidea, Holothuroidea, Ophiuroidea, Echinoidea). **A** Porcellanasteridae gen. indet.; **B** *Hymenaster* sp. indet.; **C** *Bathycrinus* sp. indet. 1; **D** *Bathycrinus* sp. indet. 2; **E** *Elpidia* sp. indet. 1 (cf *E. birsteini*); **F** *Elpidia* sp. indet. 2; **G** *Elpidia* sp. indet. 3 (cf. *E. kurilensis*); **H** *Elpidia* sp. indet. 4 (cf. *E. longicirrata*); **I** *Peniagone* spp.; **J** *Peniagone* sp. indet. 1; **K** *Peniagone* sp. indet. 2; **L** *Peniagone* sp. indet. 3; **M** *Enypniastes eximia*; **N-P** *Mesothuria* spp.; **Q** *Benthothuria* sp. indet.; **R** Ophiuroidea spp.; **S** Echinothurioida fam. indet.

The Echinodermata comprised five classes: Asteroidea, Crinoidea, Echinoidea, Holothuroidea and Ophiuroidea (Fig. 9, Table 2).

The class Asteroidea was represented by Porcellanasteridae gen. indet. (Fig. 9A, 7309-7310 m, Ryukyu Trench), *Hymenaster* sp. indet. (Fig. 9B, 6938-7369 m, Japan Trench) and a third indeterminable morphotaxon (7453 m, Japan Trench).

Two morphotaxa of crinoids (Crinoidea) were observed, present in the Japan Trench between 7121 m and 7334 m (*Bathycrinus* sp. indet. 1, Fig. 9C) and in the Izu-Ogasawara Trench between 8975 m and 9736 m (*Bathycrinus* sp. indet. 2., Fig. 9D). At the deepest base of the Boso triple junction, *Bathycrinus* sp. indet. 2 were observed in large numbers, forming dense aggregations along terraces and on rocks, as reported by Oji et al. (2009). Most individuals spread their arms in conical fans with the oral surface facing down-current, which is a typical feeding posture in near-bottom currents (Macurda and Meyer 1974).

The most diverse Echinodermata class were the Holothuroidea. Twenty morphotaxa spanning at least three orders and four families were observed (Note, there is currently no assigned family for the genus *Benthothuria*, but it is the only genus from the Persiculida order in this study and, therefore, a fourth family is assumed). The most diverse (ten morphotaxa) was the Elpidiidae family. These included four morphotaxa in the genus *Elpidia: Elpidia.* sp. indet. 1 (cf. *birsteini)* (Fig. 9E, 7001-7311 m, Japan Trench), *Elpidia* sp. indet. 2 (Fig. 9F, 7286-9037 m, Izu-Ogasawara and Japan trenches), *Elpidia* sp. indet. 3 (cf. *kurilensis)* (Fig. 9G, 8974-9047 m, Izu-Ogasawara Trench) and *Elpidia* sp. indet. 4 (cf. *longicirrata)* (Fig. 9H, 9025-9623 m, Izu-Ogasawara Trench). Three morphotaxa in the genus *Peniagone* were observed: *Peniagone* sp. indet. 1 (Fig. 9J), *Peniagone* sp. indet. 2 (Fig. 9K), *Peniagone* sp. indet. 3 (Fig. 9L). Three further morphotaxa were defined in the Elpidiidae family, but could not be confidently identified to lower taxonomic levels: Elpidiidae spp. (4913-8336 m, all trenches), *Elpidia* spp. (6819–9745 m, Izu-Ogasawara and Japan trenches) and *Peniagone* spp. (4913 -9775 m, all trenches, example shown in Fig. 9I). A further species of Elasipodida, *Enypniastes eximia* Théel 1882, was observed in all trenches between 5110 m and 6883 m, sometimes on the seafloor and, in other instances, swimming in the water overlying the seafloor (Fig. 9M). *Mesothuria* spp. (Mesothuridae) were observed in all trenches between 7109 m and 9137 m and, although morphological variation was observed (Fig. 9N-P), different morphotaxa could not be consistently identified from video data. Finally, *Benthothuria* sp. indet. (Fig. 9Q) was seen in video data from all trenches between 5932 m and 6824 m.

The only representative of the class Ophiuroidea was an indeterminable morphotype with a depth range of 4913-7339 m seen in all three trenches (Fig. 9R).

Lastly, one individual of the class Echinoidea (Echinothurioida fam. indet.) was observed and only found at 5110 m in the Ryukyu Trench (Fig. 9S). This individual occurred in the far field of view of the camera and identification is therefore limited, but video imagery was used to confirm the presence of elongate spines which it used for locomotion, characteristic of an echinoid.

### Foraminifera

**Figure 10.**
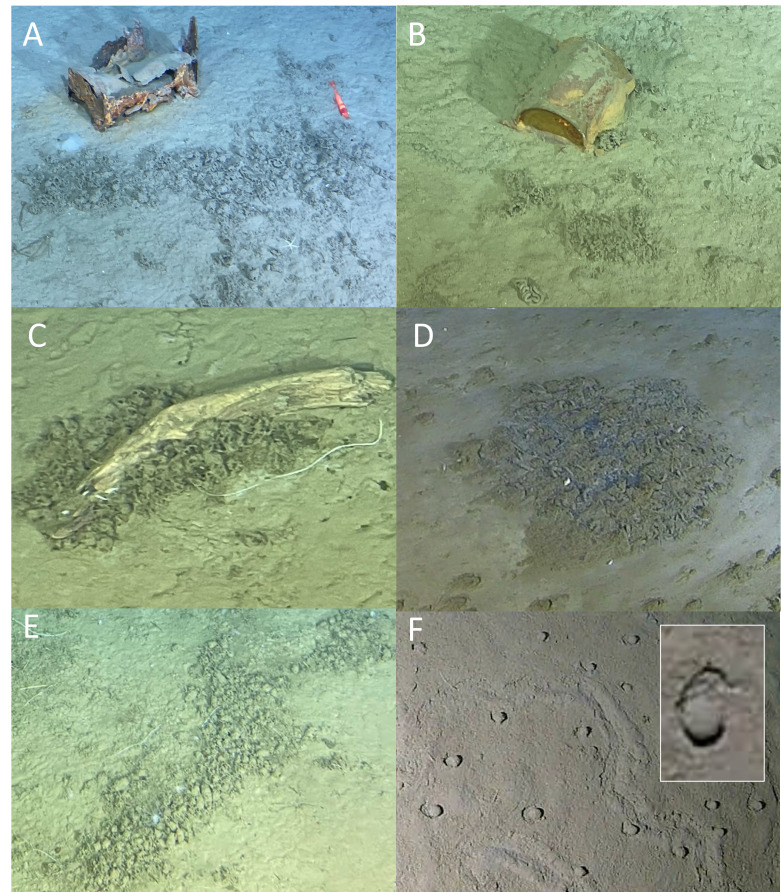
Foraminifera (Monothalamea). **A, B** Clusters associated with metal debris; **C** clusters associated with wood debris; **D** dense localised patches; **E** clusters not associated with foreign objects; **F** patch of xenophyophore-like spherical structures.

Tentatively identified foraminifera (Monothalamea) were observed in irregular patches on the seafloor, with many clusters forming around obstacles such as rocks, metallic anthropogenic debris or wood debris (Fig. 10AB, all trenches between 6938 m and 9762 m). These patches often appeared as disorganised, irregularly reticulated masses lacking any consistent pattern, possibly having disintegrated over time. The disorganised and fragmented nature of these structures makes definitive identification challenging without physical samples.

The organisms from the Izu-Ogasawara Trench, particularly near the Boso triple junction (Fig. 10C), can tentatively be classified as xenophyophores, exhibiting morphological features similar to known species such as *Shinkaiya lindsayi* (described from Japan Trench specimens at approximately 5435 m depth, Lecroq et al. (2009)) or *Syringammina* spp. (pers. comm. Andrew Gooday, NOCS, UK). These organisms also resemble presumed xenophyophores documented in seafloor imagery from the NE Atlantic at around 3000 m depth, as discussed by Tendal and Gooday (1981). The branched forms, especially those depicted in Fig. 10B, display complex, irregular reticulated structures akin to those observed in the Japan Trench, but are not always clearly associated with physical structures (Fig. 10D).

In the Ryukyu Trench, patches resembling xenophyophores were observed throughout the dive, notably in areas close to and down-current from discarded metal canisters (Fig. 10E). These patches were generally more compact and dispersed compared to those near the Boso triple junction. While some displayed irregularly reticulated patterns, many appeared fragmented or disconnected, suggesting possible disintegration or varying growth stages.

Additionally, in the Ryukyu Trench, an area roughly 2 m in diameter was noted where multiple evenly spaced, spherical “xenophyophores” were observed on the seafloor (Fig. 10F). These distinctive organisms had smooth surfaces and were surrounded by a distinct moat (see Fig. 10F inset) and may feature faint radial markings or grooves within and on parts of the margins of the moat.

### Miscellaneous

**Figure 11.**
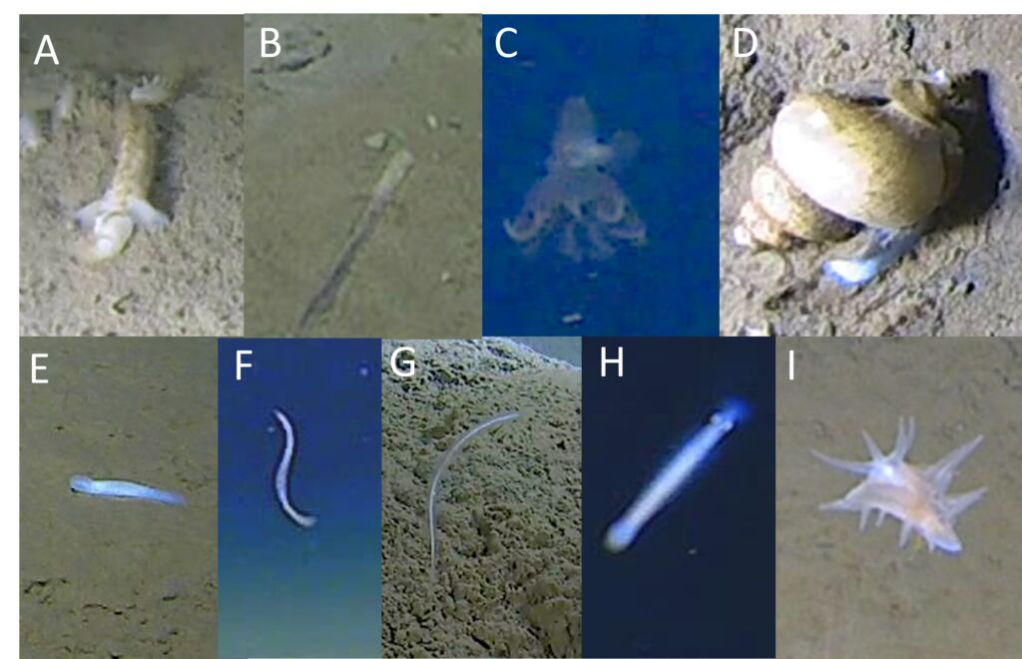
Miscellaneous (Hemichordata, Mollusca, Nemertea, Porifera, Chaetognatha, Unknown). **A** Torquaratoridae gen. indet. 1; **B** Torquaratoridae gen. indet. 2; **C** Octopoda fam. indet.; **D** Buccinidae gen. indet.; **E** Nemertea cls. indet. 1; **F** Nemertea cls. indet. 2; **G** Cladorhizidae gen. indet.; **H** Chaetognatha spp.; **I** Animalia incerta sedis.

Several phyla were represented by rare observations. These included the Hemichordata, with two enteropneust morphotaxa in the Torquatoridae family observed in the Japan Trench at 7526–7528 m (Torquatoridae gen. indet. 1, Fig. 11A) and 6819 m (Torquatoridae gen. indet. 2, Fig. 11B). Mollusca were represented by the Cephalopoda (Octopoda) and Gastropoda (Neogastropoda). A solitary individual octopod of the Cirrata suborder was seen travelling high over the bait in a lander deployment at 5941 m in the Ryukyu Trench (Fig. 11C). Deep cirrated octopods are known from similar depths, but the image quality was not sufficient to determine whether this individual was Grimpoteuthidae or Cirroteuthidae, as discussed by Jamieson and Vecchione (2022). Gastropods (Buccinidae) were seen between 5525 m and 7529 m in the Japan and Ryukyu trenches (Fig. 11D). Nemertean worms were observed crawling on the sediment surface or swimming close to the sediment surface. Two morphotaxa were observed: Nemertea cls. indet. 1 (all trenches, 4913–8291 m, Fig. 11E) and Nemertea cls. indet. 2 (Japan and Izu-Ogasawara trenches, 5445-8000 m, Fig. 11F). Nemertea cls. indet. 1 was dorsoventrally flattened and exclusively observed crawling on the sediment surface, whereas Nemertea cls. indet. 2 had an elongated body and moved using high amplitude undulations close to the sediment surface.

The only morphotaxon identified within the Porifera phylum was a demosponge belonging to the Cladorhizidae family (Fig. 11G). It was observed in the Izu-Ogasawara Trench at depths between 9568 m and 9744 m, marking the deepest in-situ observations of carnivorous sponges to date. Additionally, Chaetognatha of indeterminate class(es) (Fig. 11H) were seen in all trenches at depths ranging from 4913 m to 6870 m, swimming above the seafloor. Intriguingly, an organism of unknown phylum was recorded by a lander at 8022 m in the Japan Trench and by a submersible at 9,137 m in the Izu-Ogasawara Trench (Fig. 11I). In both instances, the organism slowly glided to the seafloor. However, due to considerations discussed below, it has not been possible to assign a definitive taxonomic classification, based on expert consensus.

## Discussion

This study utilised multiple video-based observation platforms during the 2022 “Ring of Fire Expedition” to explore abyssal and hadal biodiversity in the Japanese trenches. The results reveal a high level of diversity, although many morphotaxa remain unidentified at lower taxonomic levels, indicating that species-level diversity is likely greater. Although image-based observations did not always permit confident taxon identification, the inclusion of conservatively identified records of faunal occurrence and depth limits helps establish baseline knowledge of abyssal and hadal faunal assemblages in this region. Most morphotaxa were shared across trenches, with the Japan Trench hosting the largest number of individual morphotaxa, likely due to it also being the most extensively sampled.

### Method-specific observations

**Figure 12.**
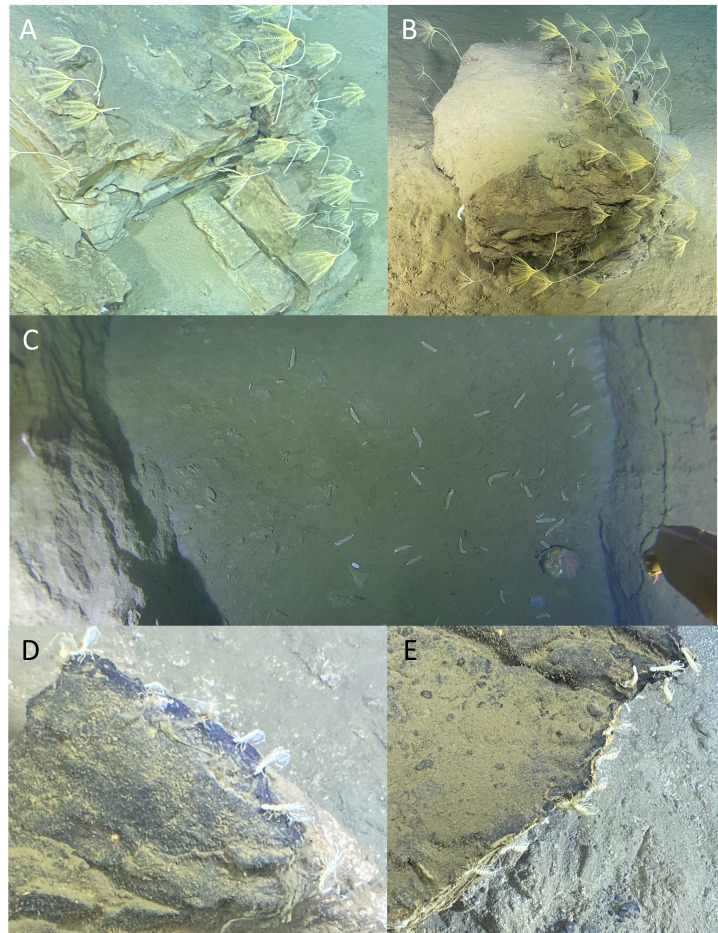
Instances of high abundance of organisms where **A** and **B** are bathycrinids aggregated in dense groups on terraces and rocks at 9137 m at the base of the Boso triple junction; **C** Echiuroidea fam. indet. 6 aggregated in fissures on the fault scarp generated by the 2011 megathrust earthquake in the Japan Trench; **D, E** Antarcturidae gen. indet. lined up along rock edges.

In addition to the established approach of baited cameras for documenting mobile fauna and exploratory vehicles for documenting sessile fauna, this study further highlights how different survey methodologies, in this case, landers and submersibles, influence both observed biodiversity and behavioural patterns. Perhaps the starkest difference are the large aggregations of scavenging amphipods that rapidly consume bait from a baited system, yet are seldom observed during a submersible dive. While baited cameras provide critical insights into mobile scavenger assemblages, they target mobile species attracted to bait. On the other hand, non-baited video transects can capture sessile and other non-bait-attending organisms across multiple habitats. In addition, larger more easily identifiable species like *Princaxelia jamiesoni* and mysids behave quite differently, depending on the observation method. Princaxelids are seen crawling and swimming in pursuit of smaller Lysianassoid amphipods when attending baited systems, but in non-baited video transects are easily identified sitting stationary on rocks (as on the left side of the rock in Fig. 12B). Likewise, mysids are often seen swimming within these aggregations of scavenging amphipods, yet from submersible observations are often seen sitting idly on the seafloor facing into the current.

These observations suggest that integrating multiple survey methods supports a holistic assessment of hadal biodiversity, as different methodologies capture different aspects of community composition and species behaviour. Comparisons with trawl sampling at these depths further underscore this. For example, Horikoshi et al. (1990) collected 6193 specimens of eight species during three trawls (two between 7420 m and 7450 m on the trench axis and one between 6700 m and 7400 m on the adjacent slope). A total of 81% of the samples collected were the bivalve *Kelliella* sp. (Venerida), although none were collected in the trawl from the slope. This suggests a significant presence of a bivalve community that is not detectable by visual assessments alone. Conversely, these trawls did not capture many of the > 100 morphotaxa from the Japan Trench in this study, except for snailfish, polynoid annelids, isopods and elpidid holothuroids, suggesting that there are easily observable sampling biases when comparing both methods.

### Patchy distributions

Other interesting observations relate to rarity and unusually high abundance of certain taxa. Two particularly striking examples of high abundance were recorded in the Izu-Ogasawara Trench. At the northern end, during a submersible dive to the base of the Boso triple junction at 9137 m in the northern end of the Izu-Ogasawara Trench, a dense aggregation of crinoids was observed – referred to as a "crinoid meadow" by Oji et al. (2009) - comprising 1524 individual crinoids attached to terraces and rocks over a 4 h transect (Fig. 12A and B). Such aggregation patterns are often attributed to localised habitat conditions, such as the presence of highly topographically complex features, including rocks and fault scarps, which provide enhanced substrate stability and facilitate attachment of sessile organisms (Oji et al. 2009). At the southern end of the Izu-Ogasawara Trench, near its deepest point, dense aggregations of cladorhizid sponges (Fig. 11G) were observed. These sponges were conspicuously absent from other surveyed locations. Their presence may reflect favourable conditions, such as elevated POC flux or fine-scale current dynamics that facilitate passive prey capture, a key factor in cladorhizid feeding ecology (Vacelet and Boury-Esnault 1995).

In contrast, substrate instability following disturbance events can also shape species aggregation patterns. An example is the dense aggregations of echiuroids on the floor of various fissures on the fault scarp generated by the 2011 Tohoku-Oki earthquake (Ueda et al. 2023; Fig. 10C). It may be that the echiuroids would have been distributed more evenly across the seafloor had they not fallen into these fissures, resulting in a usually patchy distribution, specific to this area.

Valviferan isopods (Antarcturidae gen. indet.) were only observed lined up along the acute edges of rocky outcrops (Fig. 12DE). Many valviferan isopods have a somewhat elevated anterior body that can flex to elevate the filtering setae on pereiopods 2–4 away from the substrate, a posture that is particularly pronounced in *Antarcturus* sp. indet. during feeding (Poore 2001). Based on this morphology and behaviour, we infer that the isopods may use the outcrop edges as vantage points for filter feeding, by allowing greater exposure of setae to currents or particle flow.

### Rarities and peculiarities

In the case of rarities, across the entire dataset of ~ 460 h of footage, some whole phyla or classes were represented by just one individual, for example Enteropneusta, Octopoda and Echinoidea. While acknowledging that highly mobile Octopoda may be elusive due to vehicle avoidance, as well as potentially low in abundance, the other phyla are slow-moving epibenthic groups that should exhibit no known avoidance behaviour to sampling gear and, therefore, are likely to be extremely low in abundance. Their presence is only known from collecting a large amount of footage.

**Figure 13.**
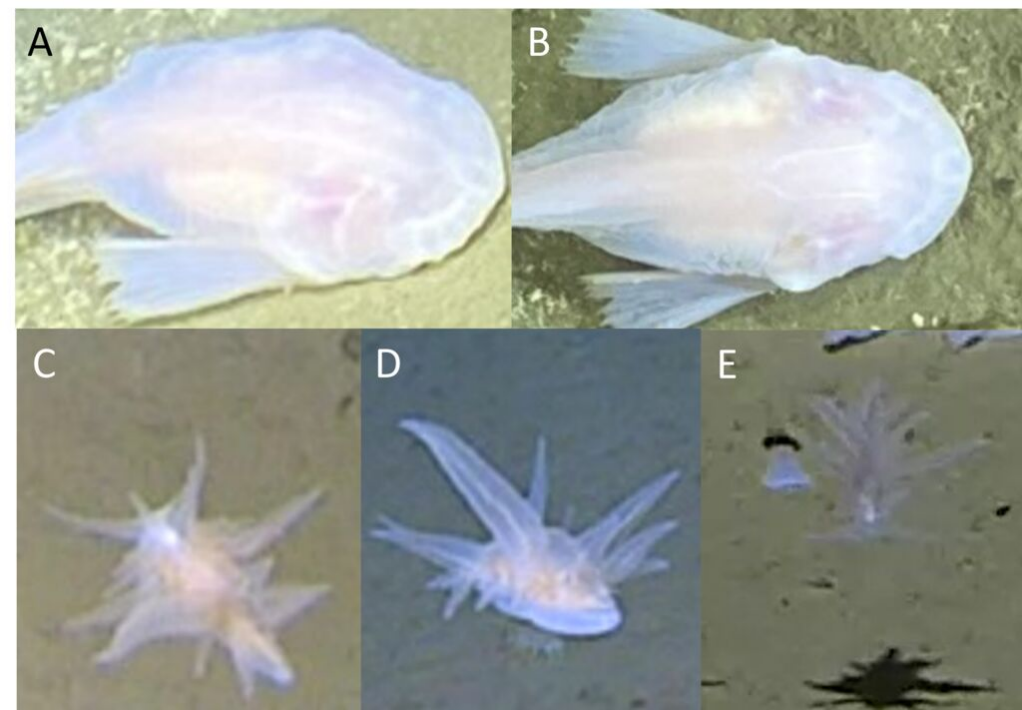
Rarities and Peculiarities. **A, B** putative eyeless snailfish (Liparidae), side view (A) and top (B); **C-E** Animalia incerta sedis, with morphological traits similar to those of a nudibranch.

In the case of both rare and peculiar, there were at least two instances of snailfish in the Japan Trench that appeared to lack eyes (Fig. 13AB). The typical black eyes of hadal snailfish are usually very prominent (see Fig. 5J-L; see Jamieson et al. (2023b) for comparison). In this instance, the individuals have body morphologies indistinguishable from *Pseudoliparis* sp. indet. 2, except for the distinct lack of eyes, nor does it appear to have visible eye sockets. Whether this is an unfortunate imaging obscurity or the result of mutation or disease, we highlight it here in the hope it can be addressed in future studies.

Another example highlighting the importance of physical sampling for definitive taxonomic classification is the identification challenge posed by an unknown metazoan of uncertain phylum, designated here as Animalia incerta sedis (Fig. 11I, Fig. 13C-E). Initially, the authors speculated that this organism might be a nudibranch. This was based on morphological features, such as bilateral symmetry, appendages akin to rhinophores, ‘leaf-like’ cerata that were longer at the anterior, similar to those of *Dirona albolineata* MacFarland, 1905 (Dironidae). In addition to the authors, several taxonomists (listed in the acknowledgements) were consulted. Some noted that the appendages appeared too rigid to belong to a nudibranch, while others speculated that they appeared to be of “molluscan morphology”, but could not speculate beyond that. Another issue regarding it being a nudibranch was that the depth of the deepest individual (9131 m) in this study was 4722 m deeper (more than double) than the next deepest known nudibranch (4435 m; Valdés (2002)). Other experts speculated that it *could* be a holothuroid, although they did not resemble any other deep-sea holothuroid, nor did it undulate in the water column in the way benthopelagic holothuroids do.

## Conclusions

This imagery-based guide, integrating video data from multiple platforms, provides a baseline for understanding megafaunal biodiversity at abyssal and hadal depths in the Japan, Ryukyu and Izu-Ogasawara trenches, with particular attention given to the 2011 earthquake epicentre site in the Japan Trench and the base of the Boso triple Junction in the Izu-Ogasawara Trench. The provided images, observational notes and species depth ranges are intended to support future campaigns by providing a reference for future biodiversity studies, including those focused on targeted specimen collection and formal taxonomic classification.

## Supplementary Material

5BE1B1F8-A2D5-52F0-A183-F49FADBA8CE0 10.3897/BDJ.14.e182172.suppl1 Supplementary material 1 Lander and submersible deployment metadata Data type Metadata File: oo_1486326.xlsx https://binary.pensoft.net/file/1486326 Jamieson, A.J.; Swanborn, D.J.B.

57DDB99A-A8AF-58B5-8264-50BBAC8CD541 10.3897/BDJ.14.e182172.suppl2 Supplementary material 2 Detailed information on biological occurrences per deployment Data type Occurrences File: oo_1517835.xlsx https://binary.pensoft.net/file/1517835 Jamieson, A.J.; Swanborn, D.J.B.

## Figures and Tables

**Table 1.** Overview of submersible dives and lander deployments conducted in the Japan, Ryukyu and Izu-Ogasawara trenches. Indicated are the number of deployments per location, and their depth ranges. Further details can be found in Suppl. material 1.

**Submersible dives**			
**Location**	**Number**	**Min Depth (m)**	**Max Depth (m)**
Japan Trench	4	7777	8002
		7465	7529
		6991	7313
		7165	7370
Ryukyu Trench	1	6937	7310
Izu-Ogasawara Trench + Boso Triple Junction	2	9559	9775
		8975	9137
**Lander deployments**			
**Location**	**Number**	**Min Depth (m)**	**Max Depth (m)**
Japan Trench	32	4913	8022
Ryukyu Trench	6	5110	7340
Izu-Ogasawara Trench + Boso Triple Junction	21	4534	9775

**Table 2.** Taxon list from the Izu-Ogasawara Trench (IOT), Japan Trench (JT) and Ryukyu Trench (RT) with locations of observations, depth ranges and reference to figures. n.s. = not shown due to low image quality.

**Taxon (Phylum - Class - Order - Family - ID)**	**Trench**	**Depth Range (m)**	**Fig. number**
Animalia incerta sedis	IOT, JPT	8022–9131	Fig. 11I
Annelida indet. 1	IOT, JPT	4534–8000	Fig. 2E
Annelida indet. 2	JPT	6579–6860	Fig. 2F
Annelida indet. 3	JPT	7259	Fig. 2G
Annelida – Echiuroidea spp.	JPT	6883–8022	ns
Annelida – Echiuroidea fam. indet. 1	JPT	6825–8022	Fig. 2A
Annelida – Echiuroidea fam. indet. 2	JPT	7528	Fig. 2B/Fig. 12C
Annelida – Echiuroidea fam. indet. 3	JPT	6883–7462	Fig. 2C
Annelida – Echiuroidea fam. indet. 4	JPT	6579	ns
Annelida – Echiuroidea fam. indet. 6	JPT	7466–7529	Fig. 2D
Annelida – Annelida spp.	IOT, JPT, RT	4913–9750	Fig. 2O
Annelida – Phyllodocida – Polynoidae – Macellicephalinae spp	IOT, JPT, RT	6665–9745	Fig. 2I
Annelida – Phyllodocida – Polynoidae – Lepidonotopodini spp	JPT, RT	7002–8001	Fig. 2J
Annelida – Phyllodocida – Polynoidae – Macellicephalinae gen. indet. 1	PT	7237–8001	Fig. 2K
Annelida – Phyllodocida – Polynoidae – Macellicephalinae gen. indet. 2	RT	7339	Fig. 2L
Annelida – Sabellida – Sabellidae spp.	IOT, JPT, RT	6991–8001	Fig. 2M
Annelida – Sabellida – Sabellidae gen. indet. 1	IOT	9026	Fig. 2N
Annelida – Terebellida – Acrocirridae spp.	IOT, JPT, RT	5531–7050	Fig. 2P
Annelida – Terebellida – Flabelligeridae spp.	JPT, RT	5941–7571	Fig. 2H
Arthropoda – Copepoda spp.	IOT, JPT	4913–7621	Fig. 3M
Arthropoda – Malacostraca – Amphipoda – Alicellidae – *Allicella gigantea*	IOT, JPT, RT	6529–8022	Fig. 3A
Arthropoda – Malacostraca – Amphipoda – Eurytheneidae – *Eurythenes* spp.	RT	6941–7310	Fig. 3B
Arthropoda – Malacostraca – Amphipoda spp.	IOT, JPT, RT	6937–9773	Fig. 3D
Arthropoda – Malacostraca – Amphipoda – Pardaliscidae – *Princaxelia jamiesoni*	IOT, JPT	7091–9742	Fig. 3C
Arthropoda – Malacostraca – Decapoda – Acanthephyridae – *Heterogenys* sp. indet. 1	IOT, JPT, RT	4534–6692	Fig. 3E
Arthropoda – Malacostraca – Decapoda – Aristeidae – *Cerataspis monstrosus*	IOT, JPT, RT	4534–6529	Fig. 3F
Arthropoda – Malacostraca – Decapoda – Benthesicymidae – *Benthesicymus crenatus*	IOT, JPT, RT	4534–7571	Fig. 3G
Arthropoda – Malacostraca – Isopoda – Antarcturidae gen. indet.	JPT	7165–7345	Fig. 3J/Fig. 12D
Arthropoda – Malacostraca – Isopoda – Munnopsidae spp.	IOT, JPT, RT	4534–9135	Fig. 3H
Arthropoda – Malacostraca – Isopoda – Munnopsidae – *Rectisura herculea*	IOT, JPT	5525–8291	Fig. 3I
Arthropoda – Malacostraca – Mysida – Mysidae spp.	IOT, JPT, RT	4534–9773	Fig. 3K
Arthropoda – Malacostraca – Tanaidacea – Gigantapseudidae – *Gigantapseudes* sp. indet.	RT	6937–7150	Fig. 3L
Chaetognatha spp.	IOT, JPT, RT	4913–6870	Fig. 11H
Chordata – Appendicularia – Copelata spp.	IOT, JPT, RT	4534–8336	Fig. 4A
Chordata – Appendicularia – Copelata – Fritillariidae spp.	IOT, JPT	5042–6579	Fig. 4C
Chordata – Appendicularia – Copelata – Oikopleuridae spp.	IOT, RT	4534–5941	Fig. 4B
Chordata – Ascidiacea spp.	JPT	7096–7345	Fig. 4D
Chordata – Ascidiacea – Phlebobranchia – Octacnemidae – *Octacnemus* sp. indet.	IOT, JPT	6006–7316	Fig. 4E
Chordata – Ascidiacea – Phlebobranchia – Octacnemidae gen. indet. 1	JPT	7241–7290	Fig. 4F
Chordata – Ascidiacea – Phlebobranchia – Octacnemidae gen. indet. 2	JPT	7170–7342	Fig. 4G
Chordata – Ascidiacea – Phlebobranchia – Octacnemidae gen. indet. 3	IOT, JPT	7345–8077	Fig. 4H
Chordata – Teleostei – Anguilliformes – Synaphobranchidae – *Ilyophis* sp. indet.	IOT	4534	Fig. 5A
Chordata – Teleostei – Beryciformes – Stephanoberycidae – *Abyssoberyx* sp. indet.	IOT	5459	Fig. 5B
Chordata – Teleostei – Gadiformes – Macrouridae – *Coryphaenoides armatus*	IOT	5042–5459	Fig. 5C
Chordata – Teleostei – Gadiformes – Macrouridae – *Coryphaenoides yaquinae*	IOT, JPT, RT	4534–7259	Fig. 5D
Chordata – Teleostei – Ophidiiformes – Ophidiidae – *Barathrites iris*	RT	5110–5941	Fig. 5E
Chordata – Teleostei – Ophidiiformes – Ophidiidae – *Bassozetus* sp. indet. 1	IOT, JPT, RT	4534–7079	Fig. 5F
Chordata – Teleostei – Ophidiiformes – Ophidiidae – *Bassozetus* sp. indet. 2	IOT, JPT, RT	5042–7079	Fig. 5G
Chordata – Teleostei – Ophidiiformes – Ophidiidae – *Bassozetus* sp. indet. 3	IOT, JPT, RT	5531–7079	Fig. 5H
Chordata – Teleostei – Ophidiiformes – Ophidiidae – *Bassozetus* sp. indet. 6	IOT, JPT, RT	5110–6529	Fig. 5I
Chordata – Teleostei – Perciformes – Liparidae – *Pseudoliparis* sp. indet. 1	IOT, JPT, RT	6023–8336	Fig. 5J
Chordata – Teleostei – Perciformes – Liparidae – *Pseudoliparis* sp. indet. 2	IOT, JPT	6579–7621	Fig. 5K
Chordata – Teleostei – Perciformes – Liparidae – *Pseudoliparis* sp. indet. 3	IOT, JPT, RT	6860–8022	Fig. 5L
Cnidaria – Hexacorallia – Anthozoa spp.	IOT, JPT, RT	7309–9745	Fig. 6I
Cnidaria – Hexacorallia – Actiniaria spp.	IOT, JPT, RT	6485–9617	n.s.
Cnidaria – Hexacorallia – Actiniaria fam. indet. 1	IOT, JPT	7528–9775	Fig. 6A
Cnidaria – Hexacorallia – Actiniaria fam. indet. 2	IOT, JPT	7526–9137	Fig. 6B
Cnidaria – Hexacorallia – Actiniaria fam. indet. 3	IOT, JPT	7095–9745	Fig. 6C
Cnidaria – Hexacorallia – Actiniaria fam. indet. 4	IOT, JPT	7082–9734	Fig. 6D
Cnidaria – Hexacorallia – Actiniaria fam. indet. 5	JPT	7180–7363	Fig. 6E
Cnidaria – Hexacorallia – Actiniaria fam. indet. 6	JPT	7527–7528	Fig. 6F
Cnidaria – Hexacorallia – Actiniaria – Galatheanthemidae – *Galatheanthemum* sp. indet. 1	JPT	7300–7500	Fig. 6H
Cnidaria – Hydrozoa – Anthoathecata – Corymorphidae gen. indet.	JPT	4913	Fig. 7C
Cnidaria – Hydrozoa – Anthoathecata – Corymorphidae – *Branchiocerianthus* sp. indet.	JPT	8001–8022	Fig. 7B
Cnidaria – Hydrozoa – Narcomedusae – Aeginidae spp.	IOT, JPT	6825–9773	Fig. 7E
Cnidaria – Hydrozoa – Narcomedusae spp.	JPT, RT	5110–8000	Fig. 7D
Cnidaria – Hydrozoa – Trachymedusae – Rhopalonematidae spp.	IOT, JPT	5459–9773	Fig. 7HM
Cnidaria – Hydrozoa – Trachymedusae – *Benthocodon* spp.	IOT, JPT	7183–9773	Fig. 7FG
Cnidaria – Hydrozoa – Trachymedusae – Rhopalonematidae – *Crossota* spp.	IOT, RT	5110–6023	Fig. 7NO
Cnidaria – Hydrozoa – Trachymedusae spp.	IOT, JPT, RT	5110–9773	n.s.
Cnidaria – Hydrozoa ord. indet.	IOT, JPT	7001–8077	n.s.
Cnidaria – Hydrozoa ord. indet. 1	IOT	9069	Fig. 7A
Cnidaria – Scyphozoa – Coronatae fam. indet.	JPT	5993	Fig. 7P
Ctenophora – cls. indet.	JPT	8000	n.s.
Ctenophora – Tentaculata – Cydippida fam. indet.	IOT, JPT, RT	4534–7519	Fig. 8EF
Ctenophora – Tentaculata – Cydippida fam. indet. 1	IOT, JPT, RT	7006–9136	Fig. 8B
Ctenophora – Tentaculata – Lobata fam. indet.	IOT, JPT, RT	5110–6177	Fig. 8A
Ctenophora – Tentaculata – Platyctenida – Lyroctenidae gen. indet.	JPT	7091–8001	Fig. 8CD
Echinodermata – Asteroidea ord. indet.	JPT	7453	n.s.
Echinodermata – Asteroidea – Paxillosida – Porcellanasteridae gen. indet.	RT	7309–7310	Fig. 9A
Echinodermata – Asteroidea – Velatida – Pterasteridae – *Hymenaster* sp. indet.	JPT	7165–7369	Fig. 9B
Echinodermata – Crinoidea – Comatulida – Bathycrinidae – *Bathycrinus* sp. indet. 1	JPT	7121–7334	Fig. 9C
Echinodermata – Crinoidea – Comatulida – Bathycrinidae – *Bathycrinus* sp. indet. 2	IOT	8975–9736	Fig. 9D/Fig. 12AB
Echinodermata – Echinoidea – Echinothurioida fam. indet.	RT	5110	Fig. 9S
Echinodermata – Holothuroidea – Elasipodida – Elpidiidae spp.	IOT, JPT, RT	4913–8336	n.s.
Echinodermata – Holothuroidea – Elasipodida – Elpidiidae – *Elpidia* spp.	IOT, JPT	6819–9745	n.s.
Echinodermata – Holothuroidea – Elasipodida – Elpidiidae – *Elpidia* sp. indet. 1 – cf. birsteini	JPT	7001–7311	Fig. 9E
Echinodermata – Holothuroidea – Elasipodida – Elpidiidae – *Elpidia* sp. indet. 2	IOT, JPT	7286–9037	Fig. 9F
Echinodermata – Holothuroidea – Elasipodida – Elpidiidae – *Elpidia* sp. indet. 3 – cf. kurilensis	IOT	8974–9047	Fig. 9G
Echinodermata – Holothuroidea – Elasipodida – Elpidiidae – *Elpidia* sp. indet. 4 – cf. longicirrata	IOT	9025–9623	Fig. 9H
Echinodermata – Holothuroidea – Elasipodida – Elpidiidae – *Peniagone* spp.	IOT, JPT, RT	4913–9775	Fig. 9I
Echinodermata – Holothuroidea – Elasipodida – Elpidiidae – *Peniagone* sp. indet. 1	JPT, RT	6937–7528	Fig. 9J
Echinodermata – Holothuroidea – Elasipodida – Elpidiidae – *Peniagone* sp. indet. 2	IOT, JPT	7168–9132	Fig. 9K
Echinodermata – Holothuroidea – Elasipodida – Elpidiidae – *Peniagone* sp. indet. 3	IOT	9680–9686	Fig. 9L
Echinodermata – Holothuroidea – Pelagothuriidae – *Enypniastes eximia*	IOT, JPT, RT	5110–6883	Fig. 9M
Echinodermata – Holothuroidea – Holothuriida – Mesothuriidae – *Mesothuria* spp.	IOT, JPT, RT	7109–9137	Fig. 9ONP
Echinodermata – Holothuroidea – Persiculida – Unassigned – *Benthothuria* sp. indet.	IOT, JPT, RT	5932–6824	Fig. 9Q
Echinodermata – Ophiuroidea spp.	IOT, JPT, RT	4913–7339	Fig. 9R
Foraminifera – Monothalamea spp.	IOT, JPT, RT	6938–9762	Fig. 10A-F
Hemichordata – Enteropneusta – Enteropneusta incertae sedis – Torquaratoridae gen. indet. 1	JPT	7526–7528	Fig. 11A
Hemichordata – Enteropneusta – Enteropneusta incertae sedis – Torquaratoridae gen. indet. 2	JPT	6819	Fig. 11B
Mollusca – Cephalopoda – Octopoda fam. indet.	RT	5941	Fig. 11C
Mollusca – Gastropoda – Neogastropoda – Buccinidae gen. indet.	JPT, RT	5525–7529	Fig. 11D
Mollusca – Gastropoda ord. indet.	JPT	7000–7370	n.s.
Nemertea cls. indet. 1	IOT, JPT, RT	4913–8291	Fig. 11E
Nemertea cls. indet. 2	IOT, JPT	5445–8000	Fig. 11F
Porifera – Demospongiae – Poecilosclerida – Cladorhizidae gen. indet.	IOT	9568–9744	Fig. 11G

## References

[B13726443] Aguzzi J., Jamieson A. J., Fujii T., Sbragaglia V., Costa C., Menesatti P., Fujiwara Y. (2012). Shifting feeding behaviour of deep-sea buccinid gastropods at natural and simulated food falls. Marine Ecology Progress Series.

[B13849991] Alldredge A. (1976). Appendicularians. Scientific American.

[B13726455] Amon D., Ziegler A., Drazen J., Grischenko A., Leitner A., Lindsay D., Voight J., Wicksten M., Young C., Smith C. (2017). Megafauna of the UKSRL exploration contract area and eastern Clarion-Clipperton Zone in the Pacific Ocean: Annelida, Arthropoda, Bryozoa, Chordata, Ctenophora, Mollusca. Biodiversity Data Journal.

[B13726470] Ando M., Tu Y., Kumagai H., Yamanaka Y., Lin C. H. (2012). Very low frequency earthquakes along the Ryukyu subduction zone. Geophysical Research Letters.

[B13726480] Bellaiche G. (1980). Sedimentation and structure of the Izu-Ogasawara (Bonin) Trench of Tokyo: New lights on the results of a diving campaign with the bathyscape “Archimede”. Earth and Planetary Science Letters.

[B13731909] Belyaev G. M. (1989). Deep-sea ocean trenches and their fauna.

[B13849983] Bone Q. (1998). The Biology of Pelagic Tunicates.

[B13735909] Bongiovanni Cassandra, Stewart Heather A., Jamieson Alan J. (2022). High‐resolution multibeam sonar bathymetry of the deepest place in each ocean. Geoscience Data Journal.

[B13731917] Buhl‐Mortensen L., Vanreusel A., Gooday A. J., Levin L. A., Priede I. G., Buhl‐Mortensen P., Gheerardyn H., King N. J., Raes M. (2010). Biological structures as a source of habitat heterogeneity and biodiversity on the deep ocean margins. Marine Ecology.

[B13731931] Elsner N. O., Malyutina M. V., Golovan O. A., Brenke N., Riehl T., Brandt A. (2015). Deep down: Isopod biodiversity of the Kuril-Kamchatka abyssal area including a comparison with data of previous expeditions of the RV Vityaz. Deep Sea Research Part II: Topical Studies in Oceanography.

[B13731942] Fujii T., Jamieson A. J., Solan M., Bagley P. M., Priede I. G. (2010). A large aggregation of liparids at 7703 m depth and a reappraisal of the abundance and diversity of hadal fish. BioScience.

[B13731952] Fukao Y., Kubota T., Sugioka H., Ito A., Tonegawa T., Shiobara H., Yamashita M., Saito T. (2021). Detection of “rapid” aseismic slip at the Izu‐Bonin Trench. Journal of Geophysical Research: Solid Earth.

[B13731974] Heezen B. C., Hollister C. D. (1971). The face of the deep.

[B13731965] Heki K., Kataoka T. (2008). On the biannually repeating slow-slip events at the Ryukyu Trench, southwestern Japan. Journal of Geophysics Research.

[B13731982] Horikoshi M., Fujita T., Ohta S. (1990). Benthic associations in bathyal and hadal depths off the Pacific coast of north eastern Japan: physiognomies and site factors. Progress in Oceanography.

[B13731991] Horton T., Marsh L., B. Bett, Gates A. R., Jones D. O., Benoist N. M., Pfeifer S., Simon-Lledó E., Durden J. M., Vandepitte L., Appeltans W. (2021). Recommendations for the standardisation of open taxonomic nomenclature for image-based identifications. Frontiers in Marine Science.

[B13732007] Howell K. L., Bullimore R. D., Foster N. L. (2014). Quality assurance in the identification of deep-sea taxa from video and image analysis: response to Henry and Roberts. ICES Journal of Marine Science.

[B13732016] Howell K. L., Davies J. S., Allcock A. L., Braga-Henriques A., Buhl-Mortensen P., Carreiro-Silva M., Dominguez-Carrió C., Durden J. M., Foster N. L., Game C. A., Hitchin B. (2019). A framework for the development of a global standardised marine taxon reference image database (SMarTaR-ID) to support image-based analyses. PloS one.

[B13732032] Itou M., Matsumura I., Noriki S. (2000). A large flux of particulate matter in the deep Japan Trench observed just after the 1994 Sanriku-Oki earthquake. Deep Sea Research Part I: Oceanographic Research Papers.

[B13732059] Jamieson A. J., Gebruk A., Fujii T., Solan M. (2011). Functional effects of the hadal sea cucumber *Elpidia
atakama* (Holothuroidea, Elasipodida) reflect small scale patterns of resource availability. Marine Biology.

[B13732068] Jamieson A. J., Fujii T., Priede I. G. (2012). Locomotory activity and feeding strategy of the hadal munnopsid isopod Rectisura
cf.
herculea (Crustacea: Asellota) in the Japan Trench. Journal of Experimental Biology.

[B13732086] Jamieson A. J., Lörz A. -N., Fujii T., Priede I. G. (2012). In situ observations of trophic behaviour and locomotion of *Princaxelia* amphipods (Crustacea, Pardaliscidae) at hadal depths in four West Pacific Trenches. Journal of the Marine Biology Association of the United Kingdom.

[B13726505] Jamieson A. J. (2018). A contemporary perspective on hadal science. Deep Sea Research Part II: Topical Studies in Oceanography.

[B13847321] Jamieson A. J., Ramsey J., Lahey P. (2019). Hadal manned submersible. Sea Technology.

[B13732097] Jamieson A. J., Linley T. D., Stewart H. A., Nargeolet P. H., Vescovo V. (2020). Revisiting the 1964 Archimède bathyscaphe dive to 7300 m in the Puerto Rico Trench, abundance of an elasipodid holothurian Peniagone sp. and a resolution to the identity of the ‘Puerto Rican snailfish’. Deep Sea Research Part I: Oceanographic Research Papers.

[B13732041] Jamieson A. J., Linley T. L. (2021). Hydrozoans, Scyphozoans, Larvaceans and Ctenophores observed in situ at hadal depths. Journal of Plankton Research.

[B13732107] Jamieson A. J., Linley T. D., Eigler S., Macdonald T. (2021). A global assessment of fishes at lower abyssal and upper hadal depths (5000 to 8000 m. Deep Sea Research Part I: Oceanographic Research Papers.

[B13732077] Jamieson A. J., Vecchione M. (2022). Hadal cephalopods: first squid observation (Oegopsida, Magnapinnidae, Magnapinna sp.) and new records of finned octopods (Cirrata) at depths> 6000 m in the Philippine trench. Marine Biology.

[B13732116] Jamieson A. J., Stewart H. A., Weston J. N.J., Lahey P., Vescovo V. L. (2022). Hadal biodiversity and potential chemosynthesis in the Java Trench. Eastern Indian Ocean. Frontiers in Marine Science.

[B13732050] Jamieson A. J., Weston J. N. (2023). Amphipoda from depths exceeding 6,000 meters revisited 60 years on. Journal of Crustacean Biology.

[B13732126] Jamieson A. J., Lindsay D., Kitazato H. (2023). Maximum depth extensions for Hydrozoa, Tunicata and Ctenophora. Marine Biology.

[B13732135] Jamieson A. J., Maroni P. J., Bond T., Niyazi Y., Kolbusz J., Arasu P., Kitazato H. (2023). New maximum depth record for bony fish: Teleostei, Scorpaeniformes, Liparidae (8336 m, Izu-Ogasawara Trench). Deep Sea Research Part I: Oceanographic Research Papers.

[B13732147] Kakui K., Fujiwara Y. (2020). First in situ observations of behavior in deep-Sea Tanaidacean Crustaceans. Zoological Science.

[B13732156] Kioka A., Schwestermann T., Moernaut J., Ikehara K., Kanamatsu T., McHugh C. M., Santos Ferreira C., Wiemer G., Haghipour N., Kopf A. J., Eglinton T. I. (2019). Megathrust earthquake drives drastic organic carbon supply to the hadal trench. Scientific Reports.

[B13732172] Komatsu T., Ceccaldi H. J. (2024). Why did the bathyscaphe FNRS III come to Japan in 1958? The beginning of French-Japanese cooperation in the field of oceanography. La Mer.

[B13732219] Komuku T., Matsumoto K., Imai Y., Sakurai T., Ito K. (2007). 1000 Dives by the Shinkai 6500 in 18 Years.. 2007 Symposium on Underwater Technology and Workshop on Scientific Use of Submarine Cables and Related Technologies.

[B13732234] Lecroq B., Gooday A. J., Tsuchiya M., Pawlowski J. (2009). A new genus of xenophyophores (Foraminifera) from Japan Trench: morphological description, molecular phylogeny and elemental analysis. Zoological Journal of the Linnean Society.

[B13732243] Lemche H., Hansen B., Madsen F. J., Tendal O. S., Wolff T. (1976). Hadal life as analysed from photographs. Videnskabelige Meddelelser Fra Dansk Naturhistorik Forening.

[B13732253] Lindsay D. J. (2005). Planktonic communities below 2000m depth. Bulletin of the Plankton Society of Japan.

[B13732262] Lindsay D. J., Miyake H. (2007). A novel benthopelagic ctenophore from 7,217 m depth in the Ryukyu Trench, Japan, with notes on the taxonomy of deepsea cydippids. Plankton and Benthos Research.

[B13732271] Lörz A. N. (2010). Trench treasures: the genus *Princaxelia* (Pardaliscidae, Amphipoda). Zoologica Baetica.

[B13732723] Macurda DB, Meyer DL (1974). Feeding posture of modern stalked crinoids. Nature.

[B13849965] Mandre Peter, Rouse Greg W. (2025). Molecular Phylogeny of the Deep-Sea Predatory Octacnemidae (Ascidiacea, Tunicata, Chordata), with Seven New Species. Diversity.

[B13732289] Markhaseva E. L., Renz J. (2025). The hadal benthopelagic Calanoida from the Kurile-Kamchatka Trench, North Pacific with the description of a new diaixid genus (Copepoda: Calanoida). Zootaxa.

[B13732321] Momma H., Watanabe M., Hashimoto K., Tashiro S. (2004). Loss of the full ocean depth ROV Kaiko-Part 1: ROV Kaiko-A review. ISOPE International Ocean and Polar Engineering Conference (ISOPE-I).

[B13732335] Nakata K., Kobayashi A., Katsumata A., Hirose F., Nishimiya T., Kimura K., Tsushima H., Maeda K., Baba H., Hanamura N., Yamada C. (2019). Double seismic zone and seismicity in the mantle wedge beneath the Ogasawara Islands identified by an ocean bottom seismometer observation. Earth, Planets and Space.

[B13847301] Nishikawa T., Ide S., Nishimura T. (2023). A review on slow earthquakes in the Japan Trench. Progress in Earth and Planetary Science.

[B13732351] Nishizawa A., Kaneda K., Oikawa M. (2009). Seismic structure of the northern end of the Ryukyu Trench subduction zone, southeast of Kyushu, Japan. Earth, Planets and Space.

[B13732360] Nishizawa A., Kaneda K., Oikawa M., Horiuchi D., Fujioka Y., Okada C. (2017). Variations in seismic velocity distribution along the Ryukyu (Nansei-Shoto) Trench subduction zone at the northwestern end of the Philippine Sea plate. Earth, Planets and Space.

[B13732371] Ogawa Y., Yanagisawa Y. (2011). Accretionary Prisms and Convergent Margin Tectonics in the Northwest Pacific Basin.

[B13732626] Oguri K., Kawamura K., Sakaguchi A., Toyofuku T., Kasaya T., Murayama M., Fujikura K., Glud R. N., Kitazato H. (2013). Hadal disturbance in the Japan Trench induced by the 2011 Tohoku-Oki Earthquake. Scientific Reports.

[B13732384] Oji T., Ogawa Y., Hunter A. W., Kitazawa K. (2009). Discovery of dense aggregations of stalked crinoids in Izu-Ogasawara Trench, Japan. Zoological Science.

[B13732393] Okutani T., Fujiwara Y. (2005). Four protobranch bivalves collected by the ROV Kaiko from hadal depths in the Japan Trench. Venus.

[B13732402] Plank T., Kelley K. A., Murray R. W., Stern L. Q. (2007). Chemical composition of sediments subducting at the Izu‐Bonin trench. Geochemistry, Geophysics, Geosystems.

[B13732411] Poore G. C. (2001). Isopoda
Valvifera: diagnoses and relationships of the families. Journal of Crustacean Biology.

[B13732420] Priede I. G., Bagley P. M., Smith A., Creasey S., Merrett N. R. (1994). Scavenging deep demersal fishes of the Porcupine Seabight, north-east Atlantic: observations by baited camera, trap and trawl. Journal of the Marine Biological Association of the United Kingdom.

[B13732430] Priede I. G., Jamieson A. J., Bond T., Kitazato H. (2024). In situ observation of a macrourid fish at 7259 m in the Japan Trench: swimbladder buoyancy at extreme depth. Journal of Experimental Biology.

[B13732439] Robison B. H., Reisenbichler K. R., Sherlock R. E. (2017). The coevolution of midwater research and ROV technology at MBARI. Oceanography.

[B13732448] Rogers A. D., Appeltans W., Assis J., Ballance L. T., Cury P., Duarte C., Favoretto F., Hynes L. A., Kumagai J. A., Lovelock C. E., Miloslavich P. (2022). Discovering marine biodiversity in the 21st century. Advances in Marine Biology.

[B13732464] Shimanaga M., Yanagi K. (2016). The Ryukyu Trench may function as a ''depocenter'' for anthropogenic marine litter. Journal of oceanography.

[B13847421] Simon-Lledó Erik, Bribiesca‐Contreras Guadalupe, Cuvelier Daphne, Durden Jennifer M., Ramalho Sofia P., Uhlenkott Katja, Arbizu Pedro Martinez, Benoist Noëlie, Copley Jonathan, Dahlgren Thomas G., Glover Adrian G., Fleming Bethany, Horton Tammy, Ju Se-Jong, Mejia-Saenz Alejandra, McQuaid Kirsty, Pape Ellen, Park Chailinn, Smith Craig R., Jones Daniel O. B., Amon Diva J. (2023). Abyssal Pacific Seafloor Megafauna Atlas. Zenodo.

[B13732473] Soh W., Taira A., Tokuyama H. (1988). A trench fan in the Izu-Ogasawara Trench on the Boso Trench triple junction, Japan. Marine Geology.

[B13847310] Stern R. J., Fouch M. J., Klemperer S. L. (2003). An overview of the Izu-Bonin-Mariana subduction factory. Geophysical Monograph-American Geophysical Union.

[B13732504] Swanborn D. J.B., Bond T., Stewart H. A., Garcia E. A., M. Stott, Kitazato H., Jamieson A. J. (2025). Seismic disturbance, productivity and depth shape hadal benthic habitats and biodiversity in the Japan, Ryukyu and Izu-Ogasawara Trenches (Northwest Pacific Ocean). Journal of Biogeography.

[B13732491] Swanborn D. J., Bond T., Kolbusz J. L., Cundy M. E., Stott M. S., Thomas E. A., Kitazato H., Jamieson A. J. (2025). Vertical zonation and environmental drivers of North-West Pacific abyssal and hadal mobile faunal communities. Deep Sea Research Part I: Oceanographic Research Papers.

[B13732482] Swan J. A., Jamieson A. J., Linley T. L., Yancey P. Y. (2021). Worldwide distribution and depth limits of decapod crustaceans (Penaeoidea, Oplophoroidea) across the abyssal-hadal transition zone of eleven subduction trenches and five additional deep-sea features. Journal of Crustacean Biology.

[B13732516] Tendal O. S., Gooday A. J. (1981). Xenophyophoria (Rhizopoda, Protozoa) in bottom photographs from the bathyal and abyssal NE Atlantic. Oceanologica Acta.

[B13732525] Ueda H., Kitazato H., Jamieson A. J., Bond T., Cardigos S., Funaki M., Maroni P., Nanbu H., O’Callaghan J. M., Onishi T., Pedersen S. S.W., Roperez J., Tsuruzono H., Watanabe H., Yasuda T. (2023). The submarine fault scarp of the 2011 Tohoku-oki earthquake in the Japan Trench. Communications Earth and Environment.

[B13732545] Vacelet J., Boury-Esnault N. (1995). Carnivorous sponges. Nature.

[B13732554] Valdés Á. (2002). Phylogenetic systematics of "*Bathydoris*" sl Bergh, 1884 (Mollusca, Nudibranchia), with the description of a new species from New Caledonian deep waters. Canadian Journal of Zoology.

[B13732563] Webb T. J., Berghe E. V., O'Dor R. (2010). Biodiversity's big wet secret: the global distribution of marine biological records reveals chronic under-exploration of the deep pelagic ocean. PLOS One.

[B13726514] Wolff T. (1960). The hadal community, an introduction. Deep Sea Research.

[B13732581] Yamazaki T., Okamura Y. (1989). Subducting seamounts and deformation of overriding forearc wedges around Japan. Tectonophysics.

